# The Examination of Viral Characteristics of HIV-1 CRF07_BC and Its Potential Interaction with Extracellular Galectin-3

**DOI:** 10.3390/pathogens9060425

**Published:** 2020-05-29

**Authors:** Chih-Yen Lin, Wen-Hung Wang, Szu-Wei Huang, Chun-Sheng Yeh, Ruei-Yu Yuan, Zih-Syuan Yang, Aspiro Nayim Urbina, Sung-Pin Tseng, Po-Liang Lu, Yen-Hsu Chen, Seng-Fan Wang

**Affiliations:** 1 Center for Tropical Medicine and Infectious Disease, Kaohsiung Medical University, Kaohsiung 80708, Taiwan; u108861002@kmu.edu.tw (C.-Y.L.); 1050101@ms.kmuh.org.tw (W.-H.W.); yeh30562@gmail.com (C.-S.Y.); kenny9517532000@gmail.com (R.-Y.Y.); aspiro.urbina@hotmail.com (A.N.U.); 830166@ms.kmuh.org.tw (P.-L.L.); d810070@kmu.edu.tw (Y.-H.C.); 2 Department of Medical Laboratory Science and Biotechnology, Kaohsiung Medical University, Kaohsiung 80708, Taiwan; u105025022@kmu.edu.tw (Z.-S.Y.); tsengsp@kmu.edu.tw (S.-P.T.); 3 Division of Infectious Disease, Department of Internal Medicine, Kaohsiung Medical, University Hospital, Kaohsiung Medical University, Kaohsiung 80708, Taiwan; 4 Model Development Section, Basic Research Laboratory, Center for Cancer Research, National Cancer Institute, Frederick, MD 20892, USA; never60814@gmail.com; 5 Department of Medical Research, Kaohsiung Medical University Hospital, Kaohsiung Medical University, Kaohsiung 80708, Taiwan

**Keywords:** Galectin-3, HIV-1, CRF07_BC, envelope, gp120, CD4

## Abstract

HIV-1 CRF07_BC is a B’ and C subtype recombinant emerging virus and many of its viral characteristics remain unclear. Galectin-3 (Gal3) is a β-galactose binding lectin that has been reported as a pattern recognition receptor (PRR) and is known to mediate adhesion between cells and microbes. This study aims to examine the viral characteristics of HIV-1 CRF07_BC virus and the role of extracellular galectin-3 in HIV-1 CRF07_BC infection. A total of 28 HIV-1+ injecting drug users (IDUs) were recruited and 24 (85.7%) were identified as HIV-1 CRF07_BC. Results indicate that significant higher serum galectin-3 was measured in CRF07_BC infected patients and CRF07_BC infection triggered significant galectin-3 expression (*p* < 0.01). Viral characteristics demonstrate that CRF07_BC virions display a higher level of envelope gp120 spikes. The virus infectivity assay demonstrated that co-treatment with galectin-3 significantly promoted CRF07_BC attachment and internalization (*p* < 0.01). A co-immunoprecipitation assay showed that pulldown galectin-3 co-precipitated both CD4 and gp120 proteins. Results from an enzyme-linked immunosorbent assay (ELISA) indicate that the galectin-3 promoting effect occurs through enhancement of the interaction between gp120 and CD4. This study suggests that CRF07_BC was predominant in HIV-1+ IDUs and CRF07_BC utilized extracellular galectin-3 to enhance its infectivity via stabilization of the gp120-CD4 interaction.

## 1. Introduction

HIV-1 CRF07_BC is a Thai B (B’) and C recombinant virus that has previously caused outbreaks in China and Taiwan [1,2]. The CRF07_BC has a mosaic pattern in its genome with a clade C backbone inserted by several clade Thai B fragments in *Gag, Pol, Env* and accessory genes [1]. It is reported that the different HIV-1 subtypes have different rates of disease progression. A previous study using meta-analysis demonstrated that the trend of disease progression among different HIV-1 subtype was subtype C > D > AE > G > A, in a descending order [3]. Patients infected with HIV-1 CRF07_BC have been reported to display slow immunological progression compared to the patients infected with subtype B [4,5]. The HIV-1 replication cycle, is initiated by attachment of virions to target cells. Attachment relies primarily on the external envelope gp120 subunit interacting with host cell CD4 receptors [6]. In a physiological setting, the binding of virion-associated gp120 to cellular CD4 is often weak and most cell types that are permissive for HIV-1 infection express low levels of CD4 [7]. Therefore, HIV-1 infection requires the assistance from various host adhesion molecules or receptors, such as dendritic cell (DC)-specific intercellular adhesion molecule 3-grabbing non-integrin binding receptor (DC-SIGN) [8] and galectin-1 [9,10,11]. 

The surface of mammalian cells, as well as, those of most enveloped viruses are heavily glycosylated. Glycans composed via specific sugar sequences presented by glycoproteins could be recognized by various glycan-binding proteins called lectins. Galectins are one of the lectin families that have been reported to play functional roles in various immune response processes through binding to host surface glycoproteins [12,13,14]. Galectins (previous called as S-type lectin) the β-galactoside binding lectins, are evolutionary conserved proteins present in many organisms. Their basic structure contains the carbohydrate-recognition domain (CRD) (about 130 amino acids), that has the ability to bind to β-galactosides, and connects with a tandem repeat domain. Currently, there are 15 galectins (galectin-1–15) that have been found in mammals so far [12]. These 15 galectins can be subdivided into three categories depending on the presentation of CRD domains including prototype, tandem-repeat and chimera [15]. The prototype galectins that have a single CRD (galectins 1, 2, 5, 7, 10, 11, 13, 14, and 15); tandem-repeat galectins, with 2 distinct but homologous CRDs (galectins 4, 6, 8, 9, and 12); and unique chimera-type, galectin 3 (Gal3), which contains a CRD fused with a large N-terminal protein-binding domain. Although galectins share similar structures, they have been reported to have different capabilities on cellular or physiological regulation. Galectin-3 has been reported to be expressed in the nucleus, the cytoplasm and the extracellular space of many cells [14,16]. Once secreted by the cell, extracellular galectin-3 plays an essential role in the processes of many fundamental physiological regulation processes [17]. In addition, galectin-3 has been reported to affect the binding of pathogens to host cells [18,19].

To date, few studies focusing on the viral characteristics of HIV-1 CRF07_BC viruses in southern Taiwan are available. Although reports indicate that patients infected with CRF07_BC displayed slow disease progression and CRF07_BC is mainly circulated among the injecting drug user (IDU) population in China and Taiwan [4,5,20], several viral characteristics of CRF07_BC remain unknown. Furthermore, there are limited reports regarding the roles of galectins in HIV-1 infection. Previous studies indicated that galectin-1 promotes HIV-1 infection through stabilization of viral attachment to macrophages [10] and CD4+ T cells [11], and galectin-9 inhibits HIV-1 infection via resistance of activated CD4+ T cells to HIV-1 infection and triggering Tim-3 downstream signaling via Gal9/Tim-3 interaction [21]. We previously demonstrated that galectin-3 promoted HIV-1 budding via association with Alix and p6^Gag^ [22]. However, these studies were mainly focused on HIV-1 subtype B, which is mainly transmitted in men who have sex with men (MSM). The envelope of HIV-1 CRF07_BC has been identified to be high glycosylated which may be beneficial in interacting with different types of galectins, such as galectin-3. Combined, this information attracted our interest in examining the viral characteristics of CRF07_BC and the potential interaction between CRF07_BC and galectin-3. Our data demonstrate that galectin-3 acts as a soluble host protein that can stabilize virus-cell interactions leading to promotion of HIV-1 CRF07_BC infection.

## 2. Results

### 2.1. CRF07_BC Was Predominant in HIV-1(+) Injecting Drug Users 

To analyze the HIV-1 genotypes of the IDU population in southern Taiwan, a cohort of HIV-1(+) IDUs, naïve of treatment, was established. Demographic results indicate that a total of 28 subjects were enrolled in this study. The average age of the subjects was 32 (range: 17–53) years old and most subjects 19 (67.9%) were within the age range of 30–49 yrs. Data from CD4 counts indicated that 27 (96.4%) patients had CD4 counts above 200 cells/mm^3^ and 5 (17.9%) of them were above 500 cells/mm^3^. Viral load data indicated that 27 (96.4%) patients had viral load below 10^5^ copies/mL and 6 (21.4%) of them were below 10^4^ copies/mL. Results from HIV-1 genotyping indicated that 3 (10.7%), 1 (3.5%), and 24 (85.7%) patients were identified as subtype B, CRF01_AE and CRF07_BC, respectively (Table 1 and Table S1).

### 2.2. Viral Characteristics of HIV-1 CRF07_BC 

**Figure 1:**
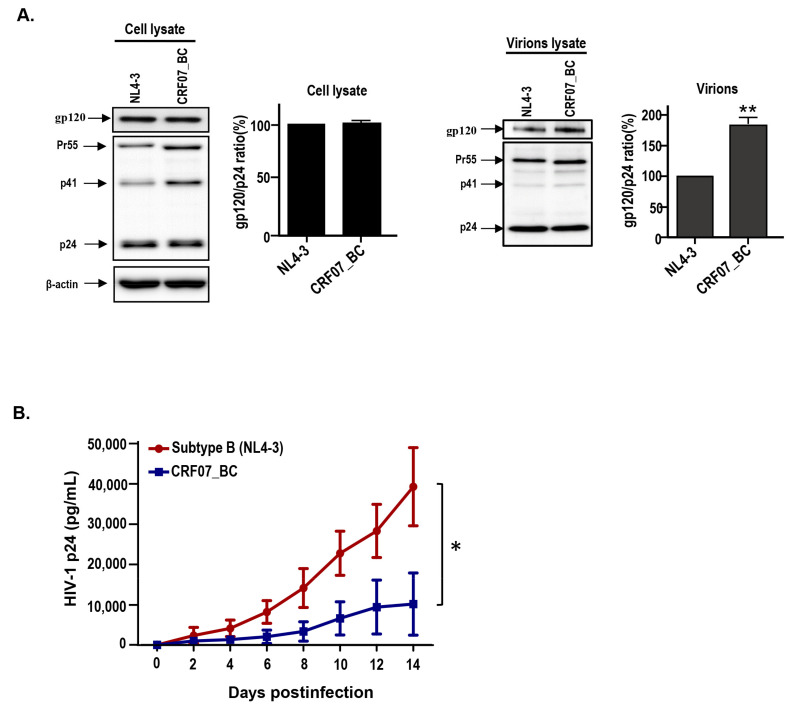
Viral characteristics of HIV-1 CRF07_BC infectious clones. The pNL4-3 and pCRF07_BC were transfected into HEK293T cells and the viral supernatants were subjected to (**A**) immunoblot analysis of cell lysates (the left) and virion lysates (the right) with indicated antibodies. Immunoblot band intensities were quantified by densitometry. The ratio of gp120/p24 was calculated based on results from the densitometry measurement. (**B**) Viral growth kinetics was conducted using phytohemagglutinin (PHA)-activated peripheral blood mononuclear cells (PBMCs), from healthy donors, infected with B subtype NL4-3 and CFR07_BC strains. The viral supernatants were collected at different time points and subjected to HIV-1 p24 determination. Results shown are the mean of four independent experiments (* *p* < 0.05; ** *p* < 0.01).

The viral characteristics of CRF07_BC were examined in this study. A HIV-1 CRF07_BC infectious clone (pCRF07_BC) was constructed using the full-length of CRF07_BC genome generated from clinical isolates (Figure S1). The full-length sequence of this infectious clone is shown in Figure S2. The HIV-1 CRF07_BC and subtype B NL4-3 viruses were generated using pCRF07_BC and pNL4-3 vectors transfected to HEK293T cells. The viral structures of CRF07_BC and subtype B NL4-3 were analyzed by a transmission electron microscope (TEM) (Figure S3A). The immunoblotting data shows that similar expression level of gp120 envelope proteins in both pNL4-3 and pCRF07_BC transfected cell lysates were detected (Figure 1A, the left) however, a significantly higher level (~1.8 folds) of gp120 expression on CRF07_BC, compared to NL4-3 viruses, was noted (*p* < 0.01) (Figure 1A, the right). Results from virus tropism analyses indicate that CRF07_BC induced cytopathic effects (CPEs) and detected budding viruses in U87-R5 cells rather than in U87-X4 cells, indicating that CRF07_BC belongs to M(R5)-tropism virus (Figure S3B,C). Results from the viral growth kinetics in monocyte-derived macrophages (MDMs) indicated that significant slower replication capabilities were noted in CRF07_BC compared to subtype B NL4-3 (*p* < 0.05) (Figure 1B). Similar results were found using Jurkat-R5 cells (Figure S4).

### 2.3. HIV-1 CRF07_BC Infection Induced Galectin-3 Expression and Secretion

**Figure 2:**
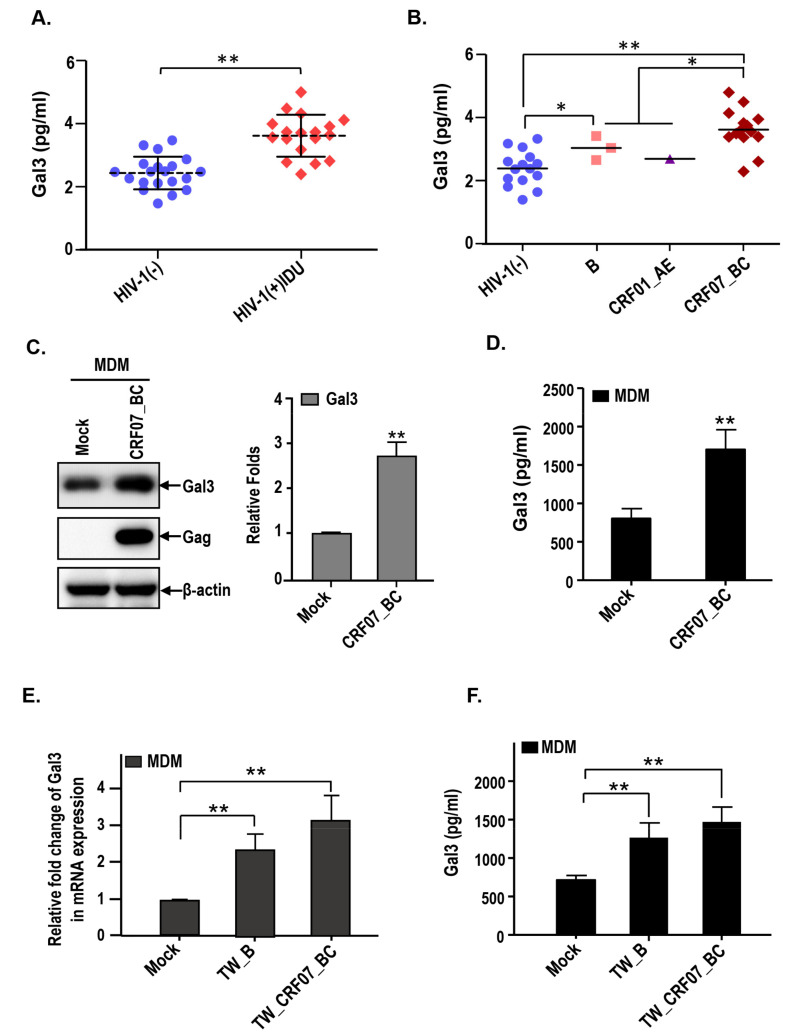
HIV-1 CRF07_BC infection induces expression and secretion of galectin-3. The serum samples from our cohort were subjected to the measurement of galectin-3 concentrations via enzyme-linked immunosorbent assay (ELISA). (**A**) The serum galectin-3 concentrations from HIV-1 (-) and HIV-1 (+) IDUs were shown. (**B**) The serum galectin-3 concentrations from HIV-1 (-) and different HIV-1 subtypes infected persons are shown. The monocytes were isolated from PBMCs and differentiated into monocyte-derived macrophages (MDMs), which were infected with HIV-1 CRF07_BC viruses. The cell lysates and supernatants were collected and subjected to (**C**) immunoblotting and (**D**) Gal3-ELISA to measure the galectin-3 expression levels. The MDMs were infected with clinical isolate, TW_B (subtype B) and TW_CRF07_BC (CRF07_BC). The cell lysates and supernatants were subjected to analyze the expression level of (**E**) galectin-3 mRNA and (**F**) secretory galectin-3 protein using quantitative real-time polymerase chain reaction (qRT-PCR) and Gal3-ELISA, respectively. Representative results are shown. Quantitative data represents the mean ± SD of results from three independent experiments (* *p* < 0.05; ** *p* < 0.01).

Next, we address the potential correlation between galectin-3 and CRF07_BC. The serum samples were collected from HIV-1(−) healthy donors and HIV-1(+) IDU groups and subjected to enzyme-linked immunosorbent assay (ELISA). The control group was selected from HIV-1(-) individuals with age and gender matching to the HIV-1(+) IDUs group. Results indicate that significantly higher serum galectin-3 in HIV-1+ IDUs, compared to the healthy control group, was detected (*p* < 0.01) (Figure 2A) and significantly higher serum galectin-3 level was measured in CRF07_BC infected patients compared to the patients infected with subtype B and CRF01_AE (Figure 2B). Furthermore, we found that HIV-1 CRF07_BC infection induced galectin-3 expression in the monocyte-derived macrophage (MDM) (Figure 2C), as well as, significantly triggered galectin-3 secretion to the culture medium compared to the mock (*p* < 0.01) (Figure 2D). This phenomenon was further confirmed by using clinical isolates, subtype B (TW_B) and CRF07_BC (TW_CRF07_BC) infected MDMs, demonstrating that TW_CRF07_BC infection induced a significantly higher galectin-3 expression detected in mRNA and supernatant proteins compared to the mock (*p* < 0.01). Similar results were observed in TW_B infected groups (Figure 2E,F).

### 2.4. Galetcin-3 Promotes HIV-1 CRF07_BC Attachment and Internalization 

**Figure 3:**
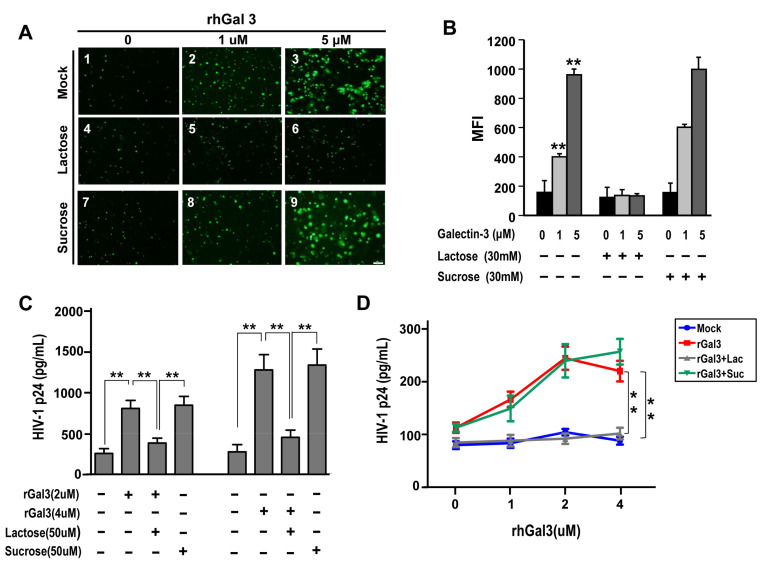
Galetcin-3 promotes HIV-1 CRF07_BC infection. (**A**) JLTRG-R5 infected with HIV-1 in the presence of rhGal3 (0–5 μM) and with/without lactose or sucrose treatment. The fluorescent cells indicate HIV-1 infected JLTRG. (**B**) The mean fluorescent index (MFI) of the JLTRG shown was (**A**) measured by flowcytometry. (**C**) Conventional viral attachment assay was performed using HIV-1 CRF07_BC viruses treated with rhGal3. Some groups were co-treated with lactose (galectin-3 inhibitor). (**D**) Conventional viral internalization assay was performed using HIV-1 CRF07_BC treated with different concentrations of rhGal3. Some groups were co-treated with lactose. All treated groups used CRF07_BC viruses, which were added to the cells together with rhGal3 with/without lactose or sucrose co-treatment. Representative results are shown. Quantitative data represent the mean ± standard deviation (SD) of results from three independent experiments (* *p* < 0.05; ** *p* < 0.01).

We further addressed whether galectin-3 plays a role in HIV-1 CRF07_BC infection. The JLTRG-R5 cells (the jurkat-CXCR4-CCR5 cells that have been stably transfected with an LTR-GFP construct) were infected with HIV-1 CRF07_BC and co-treated with/without recombinant human galectin-3 (rhGal3) then subjected to fluorescence microscope and flow cytometry analyses. Results indicated that galectin-3 co-treatment significantly increased the infectivity of CRF07_BC in JLTRG-R5 cells (*p* < 0.01). The promoting effects by galectin-3 were restricted when co-treated with lactose (galectin-3 inhibitor), as oppose to co-treatment with sucrose (Figure 3A,B). We further addressed whether galectin-3 promoted HIV-1 CRF07_BC virus attachment and internalization. Results from virus attachment assay indicated that co-treatment of galectin-3 significantly promoted CRF07_BC attaching to Jurkat-R5 cells (*p* < 0.01) and this promoting effect was observed in a dose-dependent manner (Figure 3C). Similar positive regulatory effects were produced by galectin-3 on HIV-1 CRF07_BC in virus internalization (Figure 3D). 

### 2.5. Galectin-3 Enhanced HIV-1 CRF07_BC Infection by Interacting with Viral Envelope and CD4 

**Figure 4:**
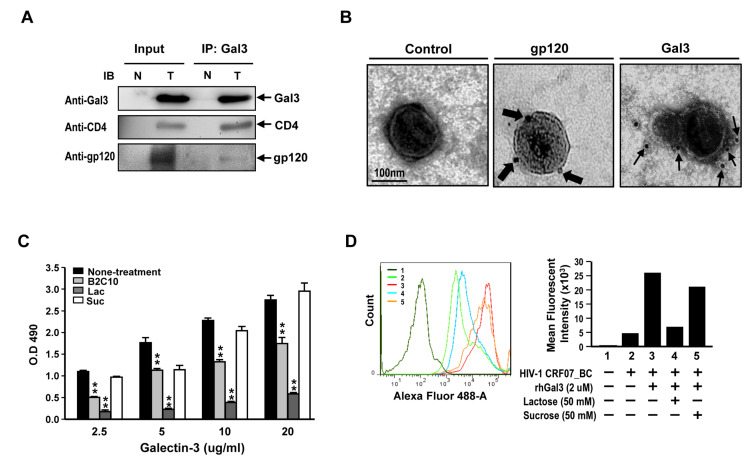
Galectin-3 interacts with HIV-1 CRF07_BC envelop gp120 and binds to the virions. (**A**) HIV-1 CRF07_BC viruses were incubated with Jurkat-R5 cells in presence of rhGal3 at 37 °C for 1 hr. The cells were collected, lysed, and immunoprecipitated with anti-Gal3 antibodies. Co-precipitated proteins were analyzed by immunoblotting for galectin-3, CD4 and gp120 envelope proteins. N and T refer to negative control (mock) and testing groups, respectively. (**B**) HIV-1 CRF07_BC viruses were incubated with galectin-3, these complexes were dropped on the grid and subjected to immuno-EM analysis with staining antibodies labeled with 6 nm or 18nm gold particles. The thin and thick arrows indicate galectin-3 and gp120 protein localization, respectively. (**C**) The HIV-1 CRF07_BC viruses coated plates were incubated with 2.5–20 (ug/mL) rhGal3 in the presence of 10 μg B2C10 monoclonal antibody, 50 mM lactose (Lac) or 50 mM sucrose (Suc). After washing with phosphate buffer saline with tween (PBST), the wells were incubated with rabbit anti-galectin-3 polyclonal antibodies and subsequently with horseradish peroxidase (HRP)-conjugated antibodies against rabbit IgG. The results were analyzed by spectrophotometer. (**D**) HIV-1 CRF07_BC viruses incubated with Jurkat-R5 cells in the presence of rhGal3, lactose or sucrose. The virus bound Jurkat-R5 cells were fixed and stained with anti-gp120 Alexa-488 labeled polyclonal antibodies and analyzed using flowcytometry. 1–5 indicate different treatments. The representative data are shown (* *p* < 0.05; ** *p* < 0.01).

To determine whether galectin-3 interact with CRF07_BC gp120 and CD4 proteins, the Jurkat-R5 cells were incubated with CRF07_BC viruses in presence of galectin-3 and the lysates were subjected to co-immunoprecipitation (Co-IP). Results indicated that CRF07_BC gp120 and CD4 proteins were co-precipitated with Gal3 (Figure 4A). Additionally, results from immuno-electron microscopy(immuno-EM), using specific antibodies to stain the HIV-1 CRF07_BC virions which were pre-treated with recombinant human galectin-3, indicated that galectin-3 bound to the envelope of CRF07_BC virions (Figure 4B). Furthermore, the binding properties of galectin-3 to HIV-1 CRF07_BC virions were validated. Results from virus-coated ELISA indicated that galectin-3 bound to CRF07_BC virions in a dose-dependent manner and the interactions would be reduced by co-treating with B_2_C_10_ (the Gal3 monoclonal antibody) and lactose (the Gal3 inhibitor) (Figure 4C). Flow cytometry data indicated that treatment of galectin-3 enhanced HIV-1 CRF07_BC viruses binding to Jurkat-R5 cells and co-treatment of lactose attenuated these promoting effects (Figure 4D). 

**Figure 5:**
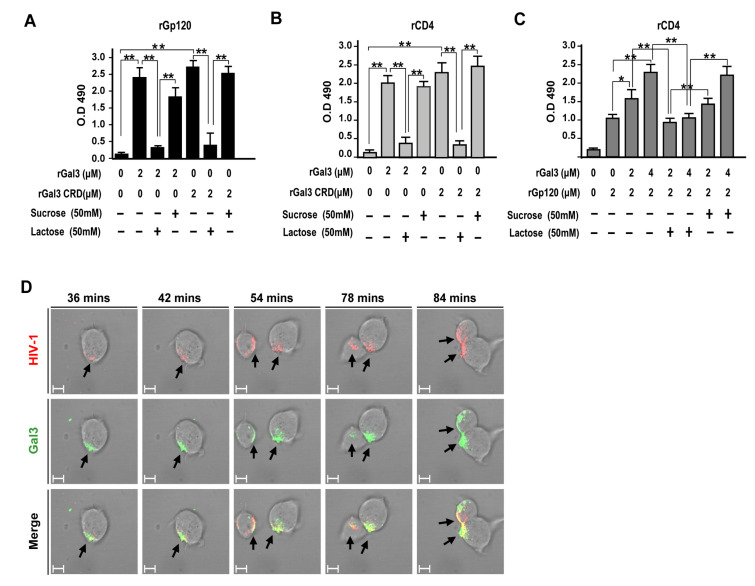
(**A**) HIV-1 CRF07_BC gp120 recombinant proteins or (**B**) CD4 recombinant proteins were coated on ELISA plates and incubated with full-length or carbohydrate-recognition domain (CRD) of galectin-3 with/without lactose or sucrose co-treatment. After washing and staining with rabbit anti-Gal3 polyclonal antibodies and adding HRP-conjugate anti-rabbit IgG, the results were analyzed using spectrophotometer. (**C**) To mimic galectin-3 promoting gp120-CD4 interaction, the CD4 recombinant proteins were coated on ELISA plates. The plates were further incubated with HIV-1 CRF07_BC gp120 recombinant proteins (2 μM) in presence of different concentrations of rhGal3 (0–4 μM). Some groups were co-treated with lactose or sucrose. After washing and staining with rabbit anti-CRF07_BC gp120 and adding HRP-conjugate anti-rabbit IgG, the results were analyzed using a spectrophotometer. (**D**) The life images of galectin-3 (Alexa-488) interaction with HIV-1 CRF07_BC (Alexa-555) on Jurkat-R5 cells were observed by time-lapse confocal microscopy (the scale bars indicate 10 um). Representative results are shown. Quantitative data represent the mean ± SD of results from three independent experiments (*p < 0.05; **p < 0.01).

Next, we examined the potential mechanism for how galectin-3 positively mediates HIV-1 CRF07_BC infection. Recombinant CRF07_BC gp120 or CD4 proteins were coated on 96-well plates and subjected to ELISA for testing of their binding properties with galectin-3. Results indicate that both galectin-3 and galectin-3 CRD significantly interacted with gp120 proteins (*p* < 0.01) and this interaction could be blocked by co-treatment with lactose as oppose to co-treatment with sucrose (Figure 5A). Similarly, we found that both galectin-3 and galectin-3 CRD significantly interacted with CD4 proteins (*p* < 0.01) and this effect was reduced when co-treated with lactose (Figure 5B). Furthermore, similar assay was preformed to investigate galectin-3 ability to enhance gp120-CD4 interaction. We found that the interaction between CRF07_BC gp120 and CD4 would be significantly improved in presence of galectin-3 (*p* < 0.01) and higher galectin-3 co-treatment promoted greater gp120-CD4 interaction (Figure 5C). Data from surface plasmon resonance (SPR) used to determine binding affinities indicated that dissociation constant (KD) of galectin-3 to interact with gp120 proteins was around 7 × 10^−9^ (M) (Figure S5). Results from time-lapse confocal microscope indicated that Alexa-488 (green fluorescent) labeled galectin-3 colocalized with Alexa-555 (red fluorescent) labeled CRF07_BC virions on the cell surface of Jurakt-R5 in a time-dependent manner (Figure 5D). 

## 3. Discussion

**Figure 6:**
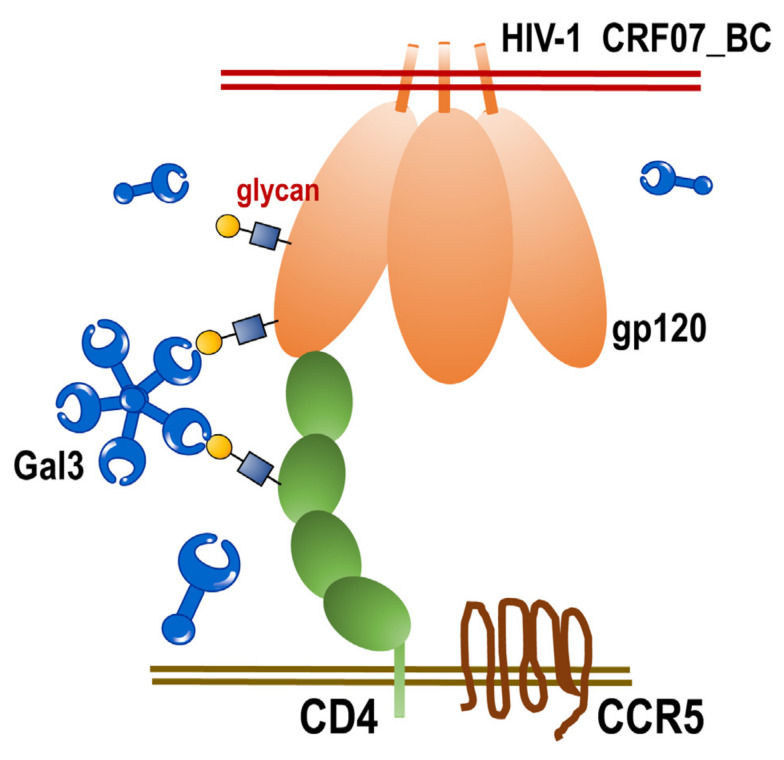
The scheme of extracellular galectin-3 promotes CRF07_BC infection. Extracellular galectin-3 binds to the glycan displayed on viral gp120 proteins and CD4 receptors. This interaction facilitates HIV-1 CRF07_BC interacting with CD4-expressing cells and enhances infectivity.

HIV-1 CRF07_BC has previously caused outbreaks in Taiwan in 2004. Many years after, this virus has been continuously transmitted among IDUs. Until now, there is still limited information regarding the various aspects of HIV-1 CRF07_BC including virus characteristics, immunoregulation, virus–host interaction and potential interaction with lectins. Galectins have been reported to participate in various cellular regulation, physiological functions, immune response and microbial infections [16,23]. In this study, we analyze the viral characteristics of HIV-1 CRF07_BC and further evaluated its interaction with galectin-3. Our data revealed that HIV-1 CRF07_BC belonged to M tropism virus, with higher levels of gp120 envelope proteins and displayed slow growth kinetics. Furthermore, we demonstrated that galectin-3 facilitated HIV-1 CRF07_BC infection, which was mainly via the CRD interacting with viral gp120 and cellular CD4 (Figure 6). To our knowledge, this is the first study to demonstrate the regulatory functions of galectin-3 on HIV-1 CRF07_BC. 

To date, the HIV-1 epidemic in Taiwan has primarily spread via sexual contact [24]. The subtype B and CRF01_AE are the cause of the majority of the infections (>90%) [25]. However, since 2003 HIV-1 CRF07_BC was gradually transmitted from China to the southern Taiwan via the heroin-trafficking route. Previous studies using molecular epidemiological survey indicate that more than 95% of IDUs with newly diagnosed HIV-1 in 2004 and 2005 were infected with CRF07_BC [26]. Similar findings and trend were found in our cohort, indicating that 85% of enrolled infected IDUs were identified as CRF07_BC infected (Table 1). Our results indicated that HIV-1 CRF07_BC is identified as a M tropism virus; however, we only used CPE and budding viruses induced by CRF07_BC specifically expressed in U87-R5 cells and, thus, additional validation is required. We suggest that further examination include the analysis of virus integration or virus production using both CCR5 and CXCR4 expressing cell lines. Furthermore, our data indicate that CRF07_BC displayed slower growth kinetics in peripheral blood mononuclear cells (PBMCs) compared to X4-tropic NL4-3 subtype B virus (Figure 1). The slow growth kinetics may correlate with slow disease progression seen by CRF07_BC patients. A previous study reported that slow immunological progression was detected in HIV-1 CRF07_BC infected injecting drug users [4]. Recently, Huang et al. reported that patients infected with CRF07_BC showed lower viral loads and higher CD4 counts [5]. These reports correspond with our data which demonstrates that 17.8% of enrolled patients had a CD4 count >500 cells/mm^3^ and 21.4% of enrolled patients had viral loads < 10^4^ copies/mL (Table 1). Taking into account this combination of factors, we suggested that the slower growth kinetics of CRF07_BC is correlated with CD4 counts, viral loads and slow immunological progression. 

Lectins are known as a unique and heterologous class of proteins with the ability to recognize and reversibly bind a variety of sugar structures present on the cell surface [27]. The broad distribution of lectins in nature show their importance in biological functions [10,28]. Here, our results indicate that higher levels of serum galectin-3 were detected in HIV-1+ IDUs compared to the healthy control, with the higher galectin-3 concentrations noted in CRF07_BC patients, compared to subtype B and CRF01_AE patients (Figure 2). Galectin-3 has been reported to be widely express in human tissues and all types of immune cell, including macrophages, monocytes, dendritic cells, eosinophils, mast cells, natural killer cells, and activated T and B cells [29,30]. We also demonstrated that CRF07_BC infection triggered galectin-3 expression and secretion (Figure 2C,D). However, this phenomenon was also observed in HIV-1 subtype B (TW_B) infected MDMs (Figure 2E,F). Thus, we suggested that other subtypes (not only CRF07_BC) may have similar inductive capabilities to trigger galectin-3 expression. Fogel et al. reported that HIV-1 Tat protein induced galectin-3 expression in CD4+ T cells [31].Therefore, we suggest that the Tat of HIV-1 CRF07_BC infection may play a role in the induction of galectin-3 expression intracellularly. Subsequently, intracellular galectin-3 are secreted out of the cells via their non-classical secretion pathway. However, the difference in induction of galectin-3 via Tat from different HIV-1 subtypes, as well as these Tat proteins binding to galectin-3 promoter to regulate galectin-3 expression in mRNA or protein level still require further investigation. 

The envelope acquired by virions occurs in the last step of the HIV-1 life cycle. However, the mechanism by which the envelope glycoprotein complex is incorporated into virus particles is still not fully understood. Viral budding efficacy is correlated to the levels of gp120 spike present on the envelope of the HIV-1 virus. Reports indicate that HIV-1 budding is rapid, and gp120-gp41 interaction are factors that contribute to relatively low levels of envelope incorporation into virus particles, leaving a residual gp120 content corresponding to ~10 trimers per virion (~10 spikes/virion) [32,33]. Our results indicate that HIV-1 CRF07_BC viruses acquired higher amounts of gp120 spikes on the envelope compared to subtype B NL4-3 viruses. Our data also indicate that CRF07_BC infection showed slow growth kinetics. Combined, we suggested that higher gp120 detected on CRF07_BC viral envelopes might occur due to slow budding. The slow budding offers enough time for a virion to acquire more inserted gp120 spikes on a cell membrane. A recent study demonstrates that HIV-1 CRF07_BC displayed a slower budding rate than subtype B NL4-3 viruses [34]. One of the possible reasons for this is that HIV-1 CRF07_BC carries amino acid deletion in p6^Gag^, which reduces Gag-Alix interaction and further results in reduction of viral budding [34]. Besides slow budding, there are other factors that may influence the gp120 acquired by viral particles, such as propagating cell types, host cell factors involved in envelope synthesis and trafficking, and different envelope incorporation ways [33]. 

In this study, our results demonstrate that galectin-3 interacted with CRF07_BC envelope gp120 and facilitated HIV-1 CRF07_BC virus attachment and internalization. Galectins bind to β-galactoside sugars, which exist in either N-linked or O-linked glycosylation [12,14,16]. These galectin recognition sugars were expressed on the HIV-1 envelope, further resulting in the interaction between the HIV-1 envelope and galectin-3. Contrary to our findings, a previous report indicated that galectin-1, instead of galectin-3, recognizes HIV-1 gp120 complex-type glycan patches and enhances HIV-1 binding to susceptible cells [9]. However, the main strain used in that study was HIV-1 subtype B. Currently, the glycosylation pattern of CRF07_BC gp120 envelope remains unclear, and we suggest that further detailed comparison and validation is necessary; owing to higher number of envelope gp120 spikes on CRF07_BC virus and difference in glycosylation pattern. In addition, it is worthy to know whether galectin-3 mediated enhancement also occurs on other types of HIV-1. 

The CRD in galectin-3 is responsible for interacting with glycans and is mainly used to regulate immune response and mediate cell-cell interaction and microbial adhesion. Our results indicate that galectin-3 binds to gp120 and CD4 proteins via the CRD, further facilitating HIV-1 CRF07_BC infection (Figure 3 and Figure 4). Galectin-3 has been reported to occur as a monomer in solution, and cross-linking via its N-terminal non-carbohydrate-binding domain only occurs upon encountering certain ligands, which appear to form dimers or higher oligomers (e.g., pentamers) [35]. Accordingly, we suggest that the CRD of galectin-3 monomers interact with HIV-1 gp120 or CD4 molecules, triggering galectin-3 oligomers formation to further enhance their binding capabilities to CD4 or gp120, respectively, thus facilitating HIV-1 CRF07_BC infection. Although the CRD is responsible for the lectin activity of galectin-3, the implication of the N-terminal domain in carbohydrate interaction still remains uncertain.

There were some limitations in this study. The sample size of the cohort was small and the study participants were not randomly selected. Accordingly, the study population might not be representative of the general population in Taiwan. Currently, we used HIV-1(-) non-IDUs as the control to be compared with HIV-1(+) IDUs. A better control group in this study would have been HIV-1(-) IDUs, however, there was a limitation in recruiting many IDUs for this study. Furthermore, regarding the comparison of serum galectin-3 levels in different HIV-1 subtypes, we only compared the differences among subtype B, CRF01_AE and CRF07_BC owing to the availability of our small cohort. The regulatory capabilities of galectin-3 to HIV-1 CRF07_BC were mainly focused on virus binding to susceptible cells (the early steps of the virus life cycle). To prove that the CRD of galectin-3 interacts with CD4 and gp120 proteins, we used full length galectin-3 and galectin-3 CRD proteins to demonstrate that both of them interact with either CD4 or gp120 proteins. Nevertheless, additional controls such as using a construct containing a different protein domain (as a negative control) or a galectin-3 with mutations in the CRD would further prove these interactions. However, due to the unavailability of these constructs or mutant proteins, we could not further validate these interactions. We also provide evidence that extracellular galectin-3 enhanced CRF07_BC infection using a recombinant human galectin-3 co-treatment strategy. To further confirm that galectin-3 mediated enhancement in CRF07_BC infection, an additional test, such as the use different cell lines, Gal3-knockdown or knockout cells and detected viral superannuates, should be considered. The roles of galectin-3 in the regulation of virus replication, budding and maturation of HIV-1 CRF07_BC are still unknown. The detailed mechanisms of how galectin-3 could be used as a potential antiviral target are worth validating.

In this study, we address the potential roles of galectin-3 in HIV-1 CRF07_BC infection. Our results concluded that galectin-3 facilitates CRF07_BC attachment and internalization via stabilization of the interaction between gp120 and CD4 and galectin-3 is suggested as a promising alternative anti-viral target. 

## 4. Materials and Methods 

### 4.1. Ethics Statement 

All study participants provided a written informed consent form. Approval was applied for and received from the Institutional Ethics Committee of the Kaohsiung Medical University, Taiwan. All procedures were performed in accordance to committee guidelines.

### 4.2. Cell Lines and Viruses 

The following cell lines were used: Jurkat-R5 CD4 T cells (Jurkat-CXCR4-CCCR5); JLTRG-R5 (Jurkat-CXCR4-CCR5 cells containing the GFP reporter gene controlled by HIV-1 LTR); U87-R5(U87 CD4^+^CCR5^+^ Cells) and U87-X4(U87 CD4^+^CXCR4^+^ Cells), and HEK293T. HIV-1 NL4-3 and CRF07_BC viruses were generated by transfecting pNL4-3 and pCRF07_BC infectious clones into HEK293 T cells and propagating in primary CD4+ T cells or PBMCs. HIV-1 CRF07_BC infectious clone was generated and the details are shown in the supplements. The titers were determined with a flow cytometry-based HIV-1 titration assay using GHOST/X4/R5 cells [36] and the details are shown in the supplement. Human PBMCs were isolated from buffy coats obtained from four healthy donors using Ficoll-Hypaque (Sigma, St. Louis, MO) gradient centrifugation. The PBMCs were activated by culture with 3 μg/mL phytohemagglutinin (PHA) in Roswell Park Memorial Institute (RPMI) 1640 medium containing 2 mM L-glutamine, 50 μg/mL gentamicin, and 10 % fetal bovine serum for 72 h. The PHA-stimulated PBMCs were used for infectivity and growth kinetic assays.

### 4.3. Infectivity, HIV-1 Genotyping Assays

JLTRG-R5, Jurkat-R5 or monocyte-derived macrophages (1 × 10^6^ /well) were used in direct infection assays. Briefly, HIV-1 viruses (multiplicity of infection (MOI) = 0.01–0.1) were incubated with these cells with/without treatment of galectin-3 at 37 °C for 48 h. Alternatively, a part of wells were co-treated with lactose (30 mM) or sucrose (30 mM). The RPMI or Dulbecco’s modified Eagle’s medium (DMEM) culture medium containing 10% serum, antibiotics, and polybrene (8 μg/mL) were used. For JLTRG-R5, HIV-1-infected cells were observed using confocal microscopy or flow cytometry. Mean fluorescent densities were measured using the Image-Pro Plus program. Regarding the HIV-1 genotyping, the protocol was according to previous publication [24]. Briefly, the blood samples were drawn from the enrolled patients. The PBMCs were purified from whole blood using Ficoll-PaqueTM density gradient centrifugation. The proviral DNA was extracted using QIAamp DNA Blood Mini Kit (Qiagen, Hilden, Germany), The gag-gene regions were amplified by polymerase chain reaction (PCR). Nested multiplex PCR was performed to determine HIV-1 subtypes. The genotype specific primers were referenced by a previous HIV-1 genotyping assay based on different sizes of amplification PCR products corresponding to each different subtype of HIV-1 [37]. To check whether the subtyping results by nested multiplex PCR were accurate, all HIV-positive samples were analyzed by sequencing and phylogenetic analysis.

### 4.4. Viral Growth Kinetic Assay

Direct infection assays were preformed utilizing Jurkat-R5-control and Jurkat-R5-Gal3 cells (1 × 10^5^ /well). Briefly, HIV-1 NL4-3 or CRF07_BC viruses (MOI = 0.1–0.01) were subjected to incubation with cells for 2 h at 37 °C in serum-free medium, followed by rinsing in phosphate-buffered saline (PBS). Subsequently, 2 mL of RPMI or DMEM medium containing 10% serum, antibiotics, and polybrene (8 μg/mL) were added, followed by incubation in 5% CO2 at 37 °C. A total of 100 μL supernatants were collected and refilled with 100 μl fresh culture medium in each well in every two days. Collected viral supernatants were subjected to HIV-1 p24 quantification using the Alliance HIV-1 P24 Antigen ELISA Kit (PerkinElmer Life and Analytical Sciences, Boston, MA; Cat. No. NEK050B).

### 4.5. Viral Attachment and Internalization Assays

The detail of viral attachment and internalization assays followed previous protocols [10,38]. Briefly, Jurkat-R5 cells (1 × 10^5^/well) were incubated with HIV-1 NL4-3 or CRF07_BC (10 ng of p24) at 4 °C for 2 h in the presence or absence of recombinant human galectin-3 (rhGal3). Some wells were co-treated with lactose (50 mM) or sucrose (50 mM). Cells were then washed with PBS three times prior to lysis with lysis buffer (PBS pH 7.2, 0.05% Tween-20, 2.5% Triton X-100 and 0.02% thimerosal). The lysates were subjected to p24 measurements using Alliance HIV-1 P24 Antigen ELISA Kit (PerkinElmer; Cat. No. NEK050B). Viral internalization assay was conducted in a similar manner, except that Jurkat-R5 cells needed to be treated with trypsin (Thermo Fisher Scientific, MA, USA) for 10 min at 37 °C to remove uninternalized viruses and washed twice with PBS prior to being lysed with the lysis buffer mentioned before. The lysates were subjected to p24 measurements. 

### 4.6. Enzyme-Linked Immunosorbent Assay (ELISA) and Co-Immunoprecipitation (Co-IP)

Virus, gp120 or CD4 recombinant protein coated ELSA and co-immunoprecipitation assays were used to evaluate the interaction between galectin-3 and CRF07_BC or galectin-3 and gp120 or gp120 and CD4. The details of these assays were shown in the supplement. 

### 4.7. Flow Cytometry and Quantitative Real-Time Polymerase Chain Reaction (qRT-PCR)

HIV-1 CRF07_BC viruses were incubated with Jurkat-R5 cells in the presence of galectin-3, lactose or sucrose at 4 °C for 1 h. After three washes with PBS, the viral bound Jurkat-R5 cells were fixed with 4% paraformaldehyde and blocked with 2% bovine serum albumin (BSA). Rabbit anti-CRF07_BC gp120 polyclonal antibodies were used to detect the binding viruses at 4 °C for 30 mins. After three washes with PBS, the viral bound cells were incubated with Alexa-488 labeled donkey anti-goat antibodies (abcam, Cat. No. ab150073) at 4 °C for 30 mins. The stained viral bound Jurkat-R5 cells were analyzed with flow cytometry. 

For measuring Gal3 mRNA expression, the quantitative real-time polymerase chain reaction (qRT-PCR) was performed. The protocol was published elsewhere [39]. Briefly, the MDMs were incubated with HIV-1 clinical isolate TW_B and TW_CRF07_BC viruses (MOI = 0.1). After incubation for 48h, the mock control and infected cells were subjected to total RNA extraction using EasyPrep Total RNA Kit (BIOTOOLS, New Taipei City, Taiwan, Cat No. DPT-BD19) and reverse transcribed using the TOOLs Easy Fast RT Kit (BIOTOOLS, Cat No. KRT-BA18), according to the manufacturer’s protocol. Real-time PCR analysis of Gal3 mRNA expression was performed using the TOOLS Easy SYBR qPCR Mix Kit (BIOTOOLS, Cat No. FPT-BB01-4) and ABI 7500 thermocycler. Primers used in qRT-PCR were as follows: human Gal-3-F (5’-GGCCACTGATTGTGCCTTAT-3’) and Gal3-R (5’TCTTTCTTCCCT TCCCCAGT-3’). Results were normalized to glyceraldehyde-3-phosphate dehydrogenase using the comparative threshold cycle method.

### 4.8. Electron Microscopy, Life Images and Surface Plasmon Resonance 

For observation of the virus morphology, galectin-3/virus interaction and affinity of galectin-3 binding to gp120, transmission electron microscopy (TEM), life image monitor, and surface plasmon resonance (SPR) were performed, respectively. The procedures were preformed according to protocols published elsewhere [22,40]. The detailed protocols are listed in the supplement. 

### 4.9. Statistical Analysis

All experiments were performed at least three times. Statistical analyses were performed using GraphPad Prism software. Statistical significance was calculated using unpaired Student’s *t*-tests (significance at *p* < 0.05). 

## Figures and Tables

**Table 1:** Characteristics of the recruited subjects.

	HIV-1(+) Injecting Drug Users (IDUs)		
Characteristics	Male (%) (*n* = 26)	Female (%) (*n* = 2)	Total (%) (*n* = 28)
**Age (yrs.)**			
15–29	8 (30.5)	1 (50)	8 (28.6)
30–49	16 (61.5)	1 (50)	19 (67.9)
≥50	2(7.6)	0 (0)	1 (3.5)
**CD4 Count (cells/mm^3^)**			
<200	1(3.8)	0(0)	1(3.5)
200–500	20(76.9)	2(100)	22(78.6)
>500	5(19.2)	0(0)	5(17.9)
**Viral Load (copies/mL)**			
<5000	1 (3.8)	0(0)	1(3.5)
5000–10,000	4(15.4)	1(50)	5 (17.9)
10,000–100,000	19(73.1)	1(50)	20(71.4)
>100,000	2(7.6)	0(0)	2(7.1)
**HIV-1 genotype**			
B	3(11.5)	0(0)	3(10.7)
CRF01_AE	0(0)	1(50)	1(3.5)
CRF07_BC	23(88.5)	1(50)	24(85.7)

## Supplementary Materials

The following are available online at https://www.mdpi.com/2076-0817/9/6/425/s1: Figure S1: Generation of full-length molecular clones. Diagram of the full-length HIV-1 CRF07_BC cloning. Figure S2. The full-length sequence of the constructed HIV-1 CRF07_BC infectious clone. Figure S3. Determination of viral characteristics of HIV-1 CRF07_BC viruses. Figure S4. Viral characteristics of HIV-1 CRF07_BC. Figure S5: Determination of the binding affinities between galectin-3 and CRF07_BC gp120.
