# Supplementary material for: The Examination of Viral Characteristics of HIV-1 CRF07_BC and Its Potential Interaction with Extracellular Galectin-3
Source: Pathogens. 2020 May 29;9(6):425. doi: 10.3390/pathogens9060425 (PMC7350312; doi:10.3390/pathogens9060425)
Supplement: Supplementary file 1 [file pathogens-09-00425-s001.pdf]

Supplementary data

Materials and Methods:

HIV-1 CRF07_BC infectious clone

The CRF07_BC isolate was collected from injected drug users (IDUs) and propagated in fresh IL-2 stimulated PBMCs. The proviral DNA was extracted using the Qiagen blood DNA kit (Qiagen, Hilden, Germany) from infected PBMCs. Specific primers were designed to amplify the full-length HIV DNA provirus as referenced [14]. The two partial molecules of the entire CRF07_BC genome were amplified with the Platinum Taq DNA polymerase High Fidelity kit (Invitrogen, Carlsberg, USA). The 5′ partial fragment and 3′ partial fragment were amplified using the primers 5LTR-F/Pol-R and Pol-F/3LTR-R, respectively. Both 5′ partial fragment and 3′ partial fragment containing the overlapping region were used as templates to generate full length CRF07_BC genome carrying SphI and EcoRV restriction sites at the 5′ and 3′ terminal, respectively, with the primers 5LTR-F/3LTR-R. The CRF07_BC full length genome was further cloned into pUC-57 vector. The morphology and infectivity of this infectious clone were validated.

Primer	Sequence	Region
5LTR-F	5′-GATAT-GGATCCTGGAAGGGCTAATTCACTCCAGGAAAA-3′	5′LTR
Pol-R	5′GTAATTCGTTAGTTTGTATGTCTGTTGCTATTATATCTATT -3′	pol
Pol-F	5′-CAGTGGCTACATGGAAGCAGAGGTTATCCCAGCAG-5′	pol
3LTR-R	5′-GCATGC-GCGGCCGCACGCGTGGTCTGAGGGATCTC-3′	3′LTR

Virus titration

HIV-1 virus titers were determined with a flow cytometry-based HIV-1 titration assay using GHOST /X4/R5 cells (GHOST are human osteosarcoma cells expressing high levels of CD4 and CCR5 and carrying LTR-GFP). The percentage of GFP expression in infected cells was measured and the Infectious Units (IU) were calculated. The virus titer was calculated by the equation- [Infectious Units (IU)/ml= (cell number) × (% of GFP-positive cells) × (dilution factor)] (The dilution factor = 1,000 μl /viral input (μl)). After the virus stocks were quantified, the experimental cells were infected at a specific multiplicity of infection (MOI). The MOI was determined via the equation: MOI = [(virus stock IU/ml) × (volume of virus used)]/(number of cells in infection). Most assays were used 10μl of CRF07_BC viral stock (10^6-10^5 IU/ml) administering to 10^5 cells/well (MOI=0.1-0.01).

ELISA

Purified HIV-1 CRF07_BC viruses (0.1 ug of HIV-1 p24/well) or glycosylated gp120 recombinant proteins (HIV-1 CRF07_BC) (0.1 ug/well) (Sino Biological, Cat. No. 11233-V08H) or recombinant human CD4 proteins (R&D Systems. Cat.No. 514-CD) were coated on 96 well plates in PBS buffer and held overnight at 4°C, blocked with 5% BSA in PBST (PBS buffer containing 0.05% Tween 20), and incubated for 1 hr at 37°C. Recombinant human galectin-3 or human galectin-3 CRD proteins (provided by GylcoCore, Institute of Biomedical Sciences, Academia Sinica, Taiwan) or recombinant gp120 proteins were added into the each well with/without co-treatment of lactose (50mM), sucrose (50mM) or B_2C_{10} monoclonal antibody (5-10 μg) at 37℃ for 2 hrs. After three washes with PBST, rabbit anti-galectin-3 polyclonal antibodies or rabbit anti-CRF07_BC gp120 polyclonal antibodies (Sino Biological, Cat. No. 11233-R011) (1:3000 dilutions) were added and incubated at 37°C for 1 hr. After three additional washes, horseradish peroxidase (HRP) conjugated sheep anti-rabbit IgG (1:3000 dilution) (Amersham Biosciences) was added prior to another 1 hr of incubation at 37°C. Finally, 200 μl of 0.015% o-phenylenediamine dihydrochloride substrate (Sigma-Aldrich, St. Louis, MO) was added before a final incubation at 37°C for

30 min. Reactions were stopped by 3N HCl and absorbances were measured with a spectrophotometer (490 nm).

Co-immunoprecipitation (Co-IP)

Jurkat-R5 cells were incubated with HIV-1 CRF07_BC viruses (200ng p24) at 37°C for 1hr in presence of rhGal3 (1μM). After three washes with PBS, cells were treated with DSP (8 μg/ml) crosslinker (3,3'-dithiodipropionic acid di[N-hydroxysuccinimide ester]) (SIGMA) at room temperature for 30 min, incubated with 1M Tris-HCl (pH 7.5) at room temperature for 15 min, and lysed in NP-40 buffer (150 mM NaCl, 25 mM Tris-HCl [pH 7.4], 1 mM EDTA, 0.5% NP-40, and 5% glycerol and containing protease inhibitor cocktail) (CALBIOCHEM). Lysates were centrifuged at 16,000 x g and supernatants were immunoprecipitated with rabbit anti-Gal3 polyclonal antibodies. Aliquots were analyzed by SDS-PAGE and immunoblotting, using antibodies against CD4, galectin-3 and CRF07_BC gp120.

Life images for monitoring galectin-3 binding with HIV-1 to T cells

HIV-1CRF07_BC viruses were labeled with Alexa-555 fluorescent dye on the envelope by using Alexa Fluor 555 carboxylic acid, succinimidyl ester (Life Technology). Recombinant galectin-3 was labeled with Alexa-488 by using Lightning-Link Conjugation kit (Innova Biosciences). Alexa-555 envelope protein labeled CRF07_BC viruses were mixed with Alexa-488 labeled galectin-3 recombinant proteins then subjected to Jurkat-R5 cells. The binding phenomenon of galectin-3 with CRF07_BC to Jurkat-R5 was observed by LSM 780 confocal microscopy.

Electron microscopy

The transmission electron microscope (TEM) was used to analyze the characteristics of the HIV-1 CRF07_BC virions and the Gal3 binding to HIV-1 CRF07_BC virions. Briefly, the concentrated and purified viral stocks were inactivated with 0.025% formaldehyde solution. One drop (~3 μL) of inactive virus in 2% glutaraldehyde buffer was applied to glow-discharged TEM copper carbon-coated grids (Electron Microscopy Sciences). To study the interaction of Gal3 with CRF07_BC virions, rGal3 (20 μg/ml) were incubated with 10ng p24 of CRF07_BC virus for 1 h at room temperature, followed by centrifugation to concentrate the complexes. The complexes were dropped onto the grids, fixed and stained with goat anti-galectin-3 (Gal3), mouse anti-gp120 (Env) or control IgG (IgG), followed by Donkey anti-goat-labeled 6-nm gold particles and Donkey anti-mouse-labeled 18-nm gold particles. Following 4% uranyl acetate staining, grids were examined by Hitachi H-7000 Transmission Electron Microscope.

Surface Plasmon Resonance (SPR)

The SPR chip used in this study was BK7 glass slide coated with a laminated Cr/Au (2/50 nm) metal layer. A mixed self-assembled monolayer (SAM) of dithiols, which consisted of 90% $C_{25}H_{44}O_6S_2$ and 10% $C_{33}H_{58}O_{11}S_2$ (SensoPath Technologies, Bozeman, MT) was used as a diluent to mix with Cr/Au (at 1:9 ratio), generating a binding matrix for the formation of the receptor monolayer (CRF07_BC gp120 protein) with the amine-coupling kit (Biacore, Inc. Uppsala, Sweden). The receptor was covalently immobilized to the SPR sensor chip according to the amine-coupling protocol. Finally, the ligands Gal3 in PBS solution were injected into the homemade flow-cell to interact with the immobilized receptor on the SPR sensor chip to form the receptor/ligand immunocomplex. To quantify the kinetic/affinity parameters between the receptor and the ligand, their interactions were measured by the surface plasmon resonance (SPR) biosensor which developed by Chou et al. [11].

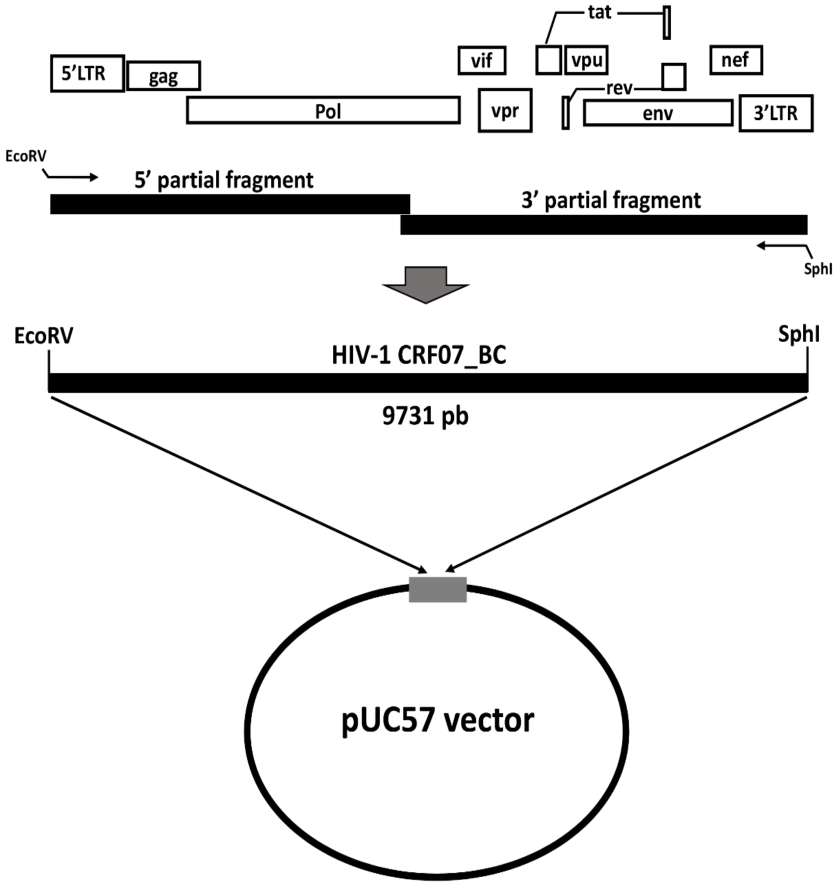

Figure S1: Generation of full-length molecular clones. Diagram of the full-length HIV-1 CRF07_BC cloning.

```
           ....|....| ....|....| ....|....| ....|....| ....|....| ....|....| ....|....| ....|....| ....|....| ....|....|
                   10         20         30         40         50         60         70         80         90        100
CRF07_BC   TGGAAGGGCT AATTCACTCC AGGAAAAGGC AAGAGATCCT TGATTTGTGG GTCTATCACA CTCAAGGCTA CTTCCCTGAT TGGCACAACT ACACACCAGG

           ....|....| ....|....| ....|....| ....|....| ....|....| ....|....| ....|....| ....|....| ....|....| ....|....|
                  110        120        130        140        150        160        170        180        190        200
CRF07_BC   ACCAGGGGTC AGATACCCAC TGACTTTTGG GTGGTGCTTC AAGCTAGTAC CAGTTGATCC AAAGGAAGTA GAAGAGGCCA ACGAAGGAGA AGACAACAGC

           ....|....| ....|....| ....|....| ....|....| ....|....| ....|....| ....|....| ....|....| ....|....| ....|....|
                  210        220        230        240        250        260        270        280        290        300
CRF07_BC   CTGCTTCACC CTGTGTGCCA GCATGGAATG GATGATGAAC ACAGAGAAGT GTTAAAGTGG AAGTTTGACA GTCACCTAGC ACACAGCCAC AAGGCCCGCC

           ....|....| ....|....| ....|....| ....|....| ....|....| ....|....| ....|....| ....|....| ....|....| ....|....|
                  310        320        330        340        350        360        370        380        390        400
CRF07_BC   AGCTACATCC GGAGTTTTAC AAAGACTGCT GACACAGAAG GGACTTTCCG CAGGGGCTTT CCACTGGGGC GTTCTGGGAG GTGTGGTCTG GGCGGGACTG

           ....|....| ....|....| ....|....| ....|....| ....|....| ....|....| ....|....| ....|....| ....|....| ....|....|
                  410        420        430        440        450        460        470        480        490        500
CRF07_BC   GGAGTGGTCA ACCCTCAGAT GCTGCATATA AGCAGCTGCT TTTCGCCTGT ACTGGGTCTC TCTAGTTAGA CCAGATCTGA GCCTGGGAGC TCTCTGGCTA

           ....|....| ....|....| ....|....| ....|....| ....|....| ....|....| ....|....| ....|....| ....|....| ....|....|
                  510        520        530        540        550        560        570        580        590        600
CRF07_BC   GCTAGGGAAC CCACTGCTTA AGCCTCAATA AAGCTTGCCT TGAGTGCTTC AAGTAGTGTG TGCCCGTCTG TTGTGTGACT CTGGTAACTA GAGATCCCTC

           ....|....| ....|....| ....|....| ....|....| ....|....| ....|....| ....|....| ....|....| ....|....| ....|....|
                  610        620        630        640        650        660        670        680        690        700
CRF07_BC   AGACCTTTTA GTCAGTGTGG AAAATCTCTA GCAGTGGCGC CCGAACAGGG ACCTGAAAGC GAAAGTAAGA CCAGAGGAGA TCTCTCGACG CAGGACTCGG

           ....|....| ....|....| ....|....| ....|....| ....|....| ....|....| ....|....| ....|....| ....|....| ....|....|
                  710        720        730        740        750        760        770        780        790        800
CRF07_BC   CTTGCTGAAA GTGCACTCGG CAAGAGGCGA GAGCGGCGAC TGGTGAGTAC GCCAATTATA TTTGACTAGC GGAGGCTAGA AGGAGAGAGA TGGGTGCGAG

           ....|....| ....|....| ....|....| ....|....| ....|....| ....|....| ....|....| ....|....| ....|....| ....|....|
                  810        820        830        840        850        860        870        880        890        900
CRF07_BC   AGCGTCAATA TTAAGAGGGG GAAAATTAGA TAAATGGGAG AAAATTAGGT TAAGGCCAGG GGGAAAGAAA CATTATATGC TAAAACATCT AGTATGGGCA


                  910        920        930        940        950        960        970        980        990       1000
CRF07_BC   AGCAGGGAGC TGGAAAGATT TGCACTTAAC CCTGGCCTTT TAGAGACATC AGAAGGCTGT AAACAAATAA TAAAACAGCT ACAACCAGCT CTTCAGACAG

           ....|....| ....|....| ....|....| ....|....| ....|....| ....|....| ....|....| ....|....| ....|....| ....|....|
                 1010       1020       1030       1040       1050       1060       1070       1080       1090       1100
CRF07_BC   GAACAGAGGA ACTTAGATCA TTATTCAACA CAGTAGCAAC TCTCTATTGT GTACATACAG AGATAGATGT ACGAGACACC AAAGAAGCCT TAGACAAGAT

           ....|....| ....|....| ....|....| ....|....| ....|....| ....|....| ....|....| ....|....| ....|....| ....|....|
                 1110       1120       1130       1140       1150       1160       1170       1180       1190       1200
CRF07_BC   AGAGGAAGAA CAAAACAAAA TTCAGCAAAA AACACAGCAG GCAAAGGAGG CTGACGGGAA GGTCAGTCAA AATTATCCTA TAGTGCAGAA TCTCCAAGGG

           ....|....| ....|....| ....|....| ....|....| ....|....| ....|....| ....|....| ....|....| ....|....| ....|....|
                 1210       1220       1230       1240       1250       1260       1270       1280       1290       1300
CRF07_BC   CAAATGGTAC ATCAGCCCTT ATCACCTAGA ACTTTAAATG CATGGGTAAA AGTGGTAGAA GAGAAGGCTT TTAGCCCAGA AGTAATACCC ATGTTTTCAG

           ....|....| ....|....| ....|....| ....|....| ....|....| ....|....| ....|....| ....|....| ....|....| ....|....|
                 1310       1320       1330       1340       1350       1360       1370       1380       1390       1400
CRF07_BC   CATTATCAGA AGGAGCCACC CCACAAGATT TAAACACCAT GCTAAACACA GTAGGGGGAC ATCAAGCAGC CATGCAAATA TTAAAAGATA CCATCAATGA

           ....|....| ....|....| ....|....| ....|....| ....|....| ....|....| ....|....| ....|....| ....|....| ....|....|
                 1410       1420       1430       1440       1450       1460       1470       1480       1490       1500
CRF07_BC   AGAGGCTGCA GAATGGGATA GATTACATCC AGTACATGCA GGGCCTATTG CACCAGGCCA AATGAGAGAA CCAAGGGGAA GTGACATAGC AGGAACTACT

           ....|....| ....|....| ....|....| ....|....| ....|....| ....|....| ....|....| ....|....| ....|....| ....|....|
                 1510       1520       1530       1540       1550       1560       1570       1580       1590       1600
CRF07_BC   AGTACCCTTC AGGAACAAAT AGCATGGATG ACAGCTAACC CACCTGTTCC AGTAGGAGAC ATCTATAAAA GATGGATAAT TCTGGGATTA AATAAAATAG

           ....|....| ....|....| ....|....| ....|....| ....|....| ....|....| ....|....| ....|....| ....|....| ....|....|
                 1610       1620       1630       1640       1650       1660       1670       1680       1690       1700
CRF07_BC   TAAGAATGTA TAGCCCTACC AGCATTCTGG ACATAAAACA AGGGCCAAAG GAACCCTTTA GAGACTATGT AGACCGGTTC TTTAAAACTT TAAGAGCGGA

           ....|....| ....|....| ....|....| ....|....| ....|....| ....|....| ....|....| ....|....| ....|....| ....|....|
                 1710       1720       1730       1740       1750       1760       1770       1780       1790       1800
CRF07_BC   ACAAGCTACA CAAGATGTAA AAAATTGGAT GACAGACACC TTGTTGGTCC AAAATGCGAA CCCAGATTGT AAGACCATTT TAAGAGCATT AGGACCAGGG

           ....|....| ....|....| ....|....| ....|....| ....|....| ....|....| ....|....| ....|....| ....|....| ....|....|
                 1810       1820       1830       1840       1850       1860       1870       1880       1890       1900
CRF07_BC   GCTTCAATAG AAGAAATGAT GACAGCATGT CAGGGAGTGG GAGGACCTAG CCATAAAGCA AGAGTGTTGG CTGAGGCAAT GAGAGATACA AACAGTACCA
```

```
            ....|....| ....|....| ....|....| ....|....| ....|....| ....|....| ....|....| ....|....| ....|....| ....|....|
                  1910       1920       1930       1940       1950       1960       1970       1980       1990       2000
CRF07_BC    TACTGATGCA GAGAAGCAAT TTTAAAAGCT CTAAAAGAGT TGTTAAATGT TTCAACTGTG GTAAGGAAGG GCACATAGCC AGAAATTGCA GGGCCCCTAG

            ....|....| ....|....| ....|....| ....|....| ....|....| ....|....| ....|....| ....|....| ....|....| ....|....|
                  2010       2020       2030       2040       2050       2060       2070       2080       2090       2100
CRF07_BC    GAAAAAGGGC TGTTGGAAAT GTGGAAAAGA AGGACACCAA ATGAAAGATT GTACTGAGAG ACAGGCCAAT TTTTTAGGGA AAATCTGGCC CTCCCACAAG

            ....|....| ....|....| ....|....| ....|....| ....|....| ....|....| ....|....| ....|....| ....|....| ....|....|
                  2110       2120       2130       2140       2150       2160       2170       2180       2190       2200
CRF07_BC    GGAAGGCCAG GGAACTTTCT TCAGAGCAGA CCAGAGCCAA CAGCCCCACC AGAGGAGAGC TTCAGGTTTG GGGAAGAGAC AACAACTCCA CCTCAGAAGC

            ....|....| ....|....| ....|....| ....|....| ....|....| ....|....| ....|....| ....|....| ....|....| ....|....|
                  2210       2220       2230       2240       2250       2260       2270       2280       2290       2300
CRF07_BC    AGGA------ ---------- -----TCCCT TAACTTCCCT CAAATCACTC TTTGGCAACG ACCCCTCGTC ACAATAAAGA TAGGGGGACA ATTAAAGGAA

            ....|....| ....|....| ....|....| ....|....| ....|....| ....|....| ....|....| ....|....| ....|....| ....|....|
                  2310       2320       2330       2340       2350       2360       2370       2380       2390       2400
CRF07_BC    GCTCTATTAG ATACAGGAGC AGATGATACA GTATTAGAAG ACATAAATTT GCCAGGGAAA TGGAAACCAA AAATGATAGG GGGAATTGGA GGTTTTATCA

            ....|....| ....|....| ....|....| ....|....| ....|....| ....|....| ....|....| ....|....| ....|....| ....|....|
                  2410       2420       2430       2440       2450       2460       2470       2480       2490       2500
CRF07_BC    AAGTAAGACA GTATGAACAG ATACCCATAG AAATCTGCGG ACACAAAGCT ATAGGTACAG TATTAGTAGG ACCTACACCT GTCAACATAA TTGGAAGAAA

            ....|....| ....|....| ....|....| ....|....| ....|....| ....|....| ....|....| ....|....| ....|....| ....|....|
                  2510       2520       2530       2540       2550       2560       2570       2580       2590       2600
CRF07_BC    TCTGTTGACT CAGCTTGGTT GCACTTTAAA TTTTCCAATC AGTCCCATTG AAACTGTACC AGTAAAATTA AAGCCAGGAA TGGATGGCCC AAAGGTTAAA

            ....|....| ....|....| ....|....| ....|....| ....|....| ....|....| ....|....| ....|....| ....|....| ....|....|
                  2610       2620       2630       2640       2650       2660       2670       2680       2690       2700
CRF07_BC    CAATGGCCAT TGACAGAAGA GAAAATAAAA GCATTAACAG AAATTTGTGA TGAAATGGAG AAGGAAGGAA AAATTACAAG AATTGGGCCT GAAAATCCAT

            ....|....| ....|....| ....|....| ....|....| ....|....| ....|....| ....|....| ....|....| ....|....| ....|....|
                  2710       2720       2730       2740       2750       2760       2770       2780       2790       2800
CRF07_BC    ATAATACTCC AATATTTGCC ATAAAAAAGA AGGACAGTAC TAAGTGGAGA AAATTAGTAG ATTTCAGGGA ACTCAATAAA AGAACTCAAG ATTTTTGGGA

            ....|....| ....|....| ....|....| ....|....| ....|....| ....|....| ....|....| ....|....| ....|....| ....|....|
                  2810       2820       2830       2840       2850       2860       2870       2880       2890       2900
CRF07_BC    AGTTCAATTA GGAATACCAC ACCCAGCAGG GTTAAAAAAG AAAAAATCAG TGACAGTACT GGATGTGGGG GATGCATATT TTTCAGTGCC TTTATATGAA

            ....|....| ....|....| ....|....| ....|....| ....|....| ....|....| ....|....| ....|....| ....|....| ....|....|
                  2910       2920       2930       2940       2950       2960       2970       2980       2990       3000
CRF07_BC    GACTTCAGGA AATATACTGC ATTCACCATA CCTAGTATAA ACAATGAAAC ACCAGGGATT AGGTATCAGT ACAATGTACT TCCGCAGGGA TGGAAAGGAT

            ....|....| ....|....| ....|....| ....|....| ....|....| ....|....| ....|....| ....|....| ....|....| ....|....|
                  3010       3020       3030       3040       3050       3060       3070       3080       3090       3100
CRF07_BC    CACCAGCAAT ATTCCAAAGT AGCATGACAA AAATCTTAGA CCCTTTTAGA AAACAAAATC CAGACATAGT TATTTATCAA TACATGGATG ATTTGTATGT

            ....|....| ....|....| ....|....| ....|....| ....|....| ....|....| ....|....| ....|....| ....|....| ....|....|
                  3110       3120       3130       3140       3150       3160       3170       3180       3190       3200
CRF07_BC    AGGATCTGAC TTAGAGATAG GGCAGCATAG AACAAAAATA GAGGAACTGA GACAACATTT GCTGAGATGG GGATTTACCA CACCAGACAA GAAACATCAG

            ....|....| ....|....| ....|....| ....|....| ....|....| ....|....| ....|....| ....|....| ....|....| ....|....|
                  3210       3220       3230       3240       3250       3260       3270       3280       3290       3300
CRF07_BC    AAAGAACCTC CATTTCTTTG GATGGGGTAT GAACTCCATC CTGACAAATG GACCGTACAA CCTATACAGC TGCCAGAAAA AGATAGCTGG ACTGTCAATG

            ....|....| ....|....| ....|....| ....|....| ....|....| ....|....| ....|....| ....|....| ....|....| ....|....|
                  3310       3320       3330       3340       3350       3360       3370       3380       3390       3400
CRF07_BC    ATATACAAAA GTTAGTGGGA AAATTAAACT GGGCAAGTCA GATTTATCCT GGAATTAAAG TAAGGCAACT TTGTAAACTC CTTAGGGGGG CCAAAGCACT

            ....|....| ....|....| ....|....| ....|....| ....|....| ....|....| ....|....| ....|....| ....|....| ....|....|
                  3410       3420       3430       3440       3450       3460       3470       3480       3490       3500
CRF07_BC    AACAGACATA GTACCGCTAA CTGAAGAAGC AGAATTAGAA TTGGCAGAAA ACAGGGAAAT TCTAAGAGAA CCAGTACATG GAGTATATTA TGACCCATCA

            ....|....| ....|....| ....|....| ....|....| ....|....| ....|....| ....|....| ....|....| ....|....| ....|....|
                  3510       3520       3530       3540       3550       3560       3570       3580       3590       3600
CRF07_BC    AAAGACTTGA TAGCTGAAAT ACAGAAACAG GGGCAGGACC AATGGACATA TCAAATTTAC CAAGAACCAT TCAAGAATCT AAAAACAGGG AAGTATGCAA

            ....|....| ....|....| ....|....| ....|....| ....|....| ....|....| ....|....| ....|....| ....|....| ....|....|
                  3610       3620       3630       3640       3650       3660       3670       3680       3690       3700
CRF07_BC    AAATGAGGAC TGCCCACACT AATGATGTAA AACAATTAAC AGAGGCTGTG CAGAAAATAG CCATGGAAAG CATAGTAATA TGGGGAAAGA CTCCTAAATT

            ....|....| ....|....| ....|....| ....|....| ....|....| ....|....| ....|....| ....|....| ....|....| ....|....|
                  3710       3720       3730       3740       3750       3760       3770       3780       3790       3800
CRF07_BC    TAGATTACCC ATCCAAAAAG AAACATGGGA GACATGGTGG ACAGACTATT GGCAAGCCAC CTGGATTCCT GAGTGGGAAT TTGTTAATAC CCCTCCCTTA

            ....|....| ....|....| ....|....| ....|....| ....|....| ....|....| ....|....| ....|....| ....|....| ....|....|
                  3810       3820       3830       3840       3850       3860       3870       3880       3890       3900
CRF07_BC    GTAAAATTAT GGTACCAGCT GGAAAAAGAT CCCATAGCAG GAGCAGAAAC TTTCTATGTA GATGGAGCAG CTAATAGGGA GACTAAAATA GGAAAAGCAG
```

```
           ....|....| ....|....| ....|....| ....|....| ....|....| ....|....| ....|....| ....|....| ....|....| ....|....|
                3910       3920       3930       3940       3950       3960       3970       3980       3990       4000
CRF07_BC   GGTATGTTAC TGACAGAGGA AGAAAGAAAG TTGTTTCTCT AACTGAAACA ACAAATCAGA AGACTGAATT GCAAGCAATT TGTATAGCTT TGCAAGATTC

           ....|....| ....|....| ....|....| ....|....| ....|....| ....|....| ....|....| ....|....| ....|....| ....|....|
                4010       4020       4030       4040       4050       4060       4070       4080       4090       4100
CRF07_BC   AGGATCAGAA GTAAACATAG TAACAGATTC ACAGTATGCA TTAGGGATCA TTCAAGCACA ACCAGATAAG AGTGAATCAG AGTTAGTTAA CCAAATAATA

           ....|....| ....|....| ....|....| ....|....| ....|....| ....|....| ....|....| ....|....| ....|....| ....|....|
                4110       4120       4130       4140       4150       4160       4170       4180       4190       4200
CRF07_BC   GAACAATTAA TAAAAAAGGA AAGAGTCTAC CTGTCATGGG TACCAGCACA TAAAGGAATT GGAGGAAATG AACAAGTAGA TAAATTAGTA AGTAATGGAA

           ....|....| ....|....| ....|....| ....|....| ....|....| ....|....| ....|....| ....|....| ....|....| ....|....|
                4210       4220       4230       4240       4250       4260       4270       4280       4290       4300
CRF07_BC   TCAGGAGAGT GCTATTTCTA GATGGAATAG ATAAAGCTCA AGAAGAGCAT GAAAGGTATC ACAGCAATTG GAGAGCAATG GCTAGTGATT TTAATCTGCC

           ....|....| ....|....| ....|....| ....|....| ....|....| ....|....| ....|....| ....|....| ....|....| ....|....|
                4310       4320       4330       4340       4350       4360       4370       4380       4390       4400
CRF07_BC   ACCAGTAGTA GCAAAAGAAA TAGTGGCTAG CTGTGATCGA TGTCAGCTAA AAGGAGAAGC CATGCATGGA CAAGTAGACT GTAGTCCAGG AATATGGCAA

           ....|....| ....|....| ....|....| ....|....| ....|....| ....|....| ....|....| ....|....| ....|....| ....|....|
                4410       4420       4430       4440       4450       4460       4470       4480       4490       4500
CRF07_BC   TTAGATTGTA CCCATTTAGA AGGAAAAATC ATCCTGGTAG CAGTCCATGT AGCCAGTGGC TACATGGAAG CAGAGGTTAT CCCAGCAGAA ACAGGACAAG

           ....|....| ....|....| ....|....| ....|....| ....|....| ....|....| ....|....| ....|....| ....|....| ....|....|
                4510       4520       4530       4540       4550       4560       4570       4580       4590       4600
CRF07_BC   AGACAGCATA CTTTATACTA AAATTAGCAG GAAGATGGCC AGTCAAAGTA ATACATACAG ACAATGGTAG TAATTTCACC AGTTCTGCAG TCAAGGCAGC

           ....|....| ....|....| ....|....| ....|....| ....|....| ....|....| ....|....| ....|....| ....|....| ....|....|
                4610       4620       4630       4640       4650       4660       4670       4680       4690       4700
CRF07_BC   CTGTTGGTGG GCAGGTATCC AACAGGAATT TGGAATTCCC TACAATCCCC AAAGTCAGGG AGTAGTAGAA TCCATGAATA AAGAATTAAA GAAAATTATA

           ....|....| ....|....| ....|....| ....|....| ....|....| ....|....| ....|....| ....|....| ....|....| ....|....|
                4710       4720       4730       4740       4750       4760       4770       4780       4790       4800
CRF07_BC   GGGCAGGTAA GAGATCAAGC TGAGCACCTT AAGACAGCAG TACAAATGGC AGTATTCATT CACAATTTTA AAAGAAAAGG GGGGATTGGG GGGTACAGTG

           ....|....| ....|....| ....|....| ....|....| ....|....| ....|....| ....|....| ....|....| ....|....| ....|....|
                4810       4820       4830       4840       4850       4860       4870       4880       4890       4900

CRF07_BC   CAGGGGAAAG AATAATAGAT ATAATAGCAA CAGACATACA AACTAACGAA TTACAAAAAC AAATTACAAA AATTCAAAAT TTTCGGGTTT ATTACAGAGA

           ....|....| ....|....| ....|....| ....|....| ....|....| ....|....| ....|....| ....|....| ....|....| ....|....|
                4910       4920       4930       4940       4950       4960       4970       4980       4990       5000
CRF07_BC   CAGCAGAGAC CCCAGTTGGA AAGGACCAGC CAAACTACTC TGGAAAGGTG AAGGGGCAGT AGTAATACAA GATAATAGTG ACATAAAGGT AGTACCAAGG

           ....|....| ....|....| ....|....| ....|....| ....|....| ....|....| ....|....| ....|....| ....|....| ....|....|
                5010       5020       5030       5040       5050       5060       5070       5080       5090       5100
CRF07_BC   AGGAAAGCAA AAATCATTAA GGACTATGGA AAACAGATGG CAGGTGCTGA TTGTGTGGCA GGTAGACAGG ATGAAGATTA GAACATGGAA TAGTTTAGTA

           ....|....| ....|....| ....|....| ....|....| ....|....| ....|....| ....|....| ....|....| ....|....| ....|....|
                5110       5120       5130       5140       5150       5160       5170       5180       5190       5200
CRF07_BC   AAACACCATA TGTATGTTTC AAAGAGAGCT AATGGATGGT TTTACAGACA TCATTATGAA AGCAGACATC CAAAAGTAAG TTCAGAAGTA CACATCCCAT

           ....|....| ....|....| ....|....| ....|....| ....|....| ....|....| ....|....| ....|....| ....|....| ....|....|
                5210       5220       5230       5240       5250       5260       5270       5280       5290       5300
CRF07_BC   TAGGAGAGGC TAAATTAGTA ATAAAAACAT ATTGGGGGTT GCAAACAGGA GAAAGAGATT GGCATTTGGG TCATGGAGTC TCCATAGAAT GGAGATTGAG

           ....|....| ....|....| ....|....| ....|....| ....|....| ....|....| ....|....| ....|....| ....|....| ....|....|
                5310       5320       5330       5340       5350       5360       5370       5380       5390       5400
CRF07_BC   AAGATATACC ACACAAATAG AACCTGGCCT GGCAGACCAG CTAATTCATT TGTATTATTT TGATTGTTTT GCAGACTCTG CTATAAGGAA AGCCATATTA

           ....|....| ....|....| ....|....| ....|....| ....|....| ....|....| ....|....| ....|....| ....|....| ....|....|
                5410       5420       5430       5440       5450       5460       5470       5480       5490       5500
CRF07_BC   GGACACATAG TTATTCCTAG GTGTGACTAT CAAGCAGGAC ATAATAATAA GGTAGGATCT CTACAATATT TGGCACTGAC AGCATTGATA AAACCAAGAA

           ....|....| ....|....| ....|....| ....|....| ....|....| ....|....| ....|....| ....|....| ....|....| ....|....|
                5510       5520       5530       5540       5550       5560       5570       5580       5590       5600
CRF07_BC   AGACAAAGCC ACCTCTGCCT AGTATTAAGA AATTAGTAGA GGATAGATGG AACAATCCCC AGAAGATCAG GGGCCACAGA GGGAACCATA CAATGAATGG

           ....|....| ....|....| ....|....| ....|....| ....|....| ....|....| ....|....| ....|....| ....|....| ....|....|
                5610       5620       5630       5640       5650       5660       5670       5680       5690       5700
CRF07_BC   ACACTAGAGC TTCTAGAAGA GCTCAAGCAG GAAGCTGTCA GACACTTTCC TAGACCATGG CTTCATGGCT TAGGACAACA TATCTATGAA ACATATGGGG

           ....|....| ....|....| ....|....| ....|....| ....|....| ....|....| ....|....| ....|....| ....|....| ....|....|
                5710       5720       5730       5740       5750       5760       5770       5780       5790       5800
CRF07_BC   ATACTTGGGC AGGAGTGGAA GCCATAATAA GAATTCTGCA ACAACTGCTG TTTATTCATT TCAGAATTGG GTGTCAGCAT AGCAGAATAG GCATTTTGAG
```

```
                 5810       5820       5830       5840       5850       5860       5870       5880       5890       5900
CRF07_BC   ACAGAGAAGA ACAAGAAATG GAGCCAGTAG ATCCTAATTT AGAGCCTTGG AAGCATCCAG GAAGTCAGCC TAAGACTGCT TGTACCAATT GCTATTGTAA

           ....|....| ....|....| ....|....| ....|....| ....|....| ....|....| ....|....| ....|....| ....|....| ....|....|
                 5910       5920       5930       5940       5950       5960       5970       5980       5990       6000
CRF07_BC   AAAGTGCTGC TTTCATTGCC AAGTTTGTTT CATGCAAAAA GGCTTAGGCA TCTTCTATGG CAGGAAGAAG CGAAGAAAAC GACGACGAGC TCCTCAGAGC

           ....|....| ....|....| ....|....| ....|....| ....|....| ....|....| ....|....| ....|....| ....|....| ....|....|
                 6010       6020       6030       6040       6050       6060       6070       6080       6090       6100
CRF07_BC   AGTGAGGATC ATCAAGATCT TATATCAAAG CAGTAAGTAG TAAATGTAAT GCAAGCTTTA ATCGTTTTAG CAATAGTAGC CTTAGTAGTA GTAGCAATAA

           ....|....| ....|....| ....|....| ....|....| ....|....| ....|....| ....|....| ....|....| ....|....| ....|....|
                 6110       6120       6130       6140       6150       6160       6170       6180       6190       6200
CRF07_BC   TAGCAATAGT TGTGTGGACC ATAGTATTCA TAGAATATAG GAAAATATTA AGACAGAGAA AAATAGACAG GTTAATTGAT AGAATAAGAG AAAGAGCAGA

           ....|....| ....|....| ....|....| ....|....| ....|....| ....|....| ....|....| ....|....| ....|....| ....|....|
                 6210       6220       6230       6240       6250       6260       6270       6280       6290       6300
CRF07_BC   AGACAGTGGC AATGAGAGTG ACGGGGATCA GGAAGAATTA TCGGCATTTA TGGGGATGGG GCACCATGCT CCTTGGGATG TTGATGATCT GTAGTGTTGT

           ....|....| ....|....| ....|....| ....|....| ....|....| ....|....| ....|....| ....|....| ....|....| ....|....|
                 6310       6320       6330       6340       6350       6360       6370       6380       6390       6400
CRF07_BC   AGGAAACTTC TGGGTCACAG TCTATTATGG GGTACCTGTA TGGAAAGAAG CAACCACCAC TTTATTTTGT GCATCAGATG CTAAAGCATA TGATACAGAG

           ....|....| ....|....| ....|....| ....|....| ....|....| ....|....| ....|....| ....|....| ....|....| ....|....|
                 6410       6420       6430       6440       6450       6460       6470       6480       6490       6500
CRF07_BC   GTACATAATG TTTGGGCTAC ACATGCCTGT GTACCCACAG ACCCCAACCC ACAAGAAATG GTTTTGGAAA ATGTAACAGA AAATTTTAAC ATGTGGAAAA

           ....|....| ....|....| ....|....| ....|....| ....|....| ....|....| ....|....| ....|....| ....|....| ....|....|
                 6510       6520       6530       6540       6550       6560       6570       6580       6590       6600
CRF07_BC   ATGAAATGGT AAATCAGATG CACGAAGATG TAATCAGTTT ATGGGATCAA AGCCTAAAGC CATGTGTAAA GTTGACCCCA CTCTGTGTCA CTTTAGAATG

           ....|....| ....|....| ....|....| ....|....| ....|....| ....|....| ....|....| ....|....| ....|....| ....|....|
                 6610       6620       6630       6640       6650       6660       6670       6680       6690       6700
CRF07_BC   TAGAAATGTT ACCCGCAATG AGAGTGTGAG AAATGTTACC CACAATGAGA GCGGGAATGG AATGAAAAAT TGCTCTTTCA ATGCAACCAC AGTAGTAAGA

           ....|....| ....|....| ....|....| ....|....| ....|....| ....|....| ....|....| ....|....| ....|....| ....|....|
                 6710       6720       6730       6740       6750       6760       6770       6780       6790       6800
CRF07_BC   GATAGGCAGC AGAAAGTGTA TGCACTTTTT TATAGACTTG ATATAGTACC ACTTACTAAT AATAAGAACT CTAGTGAGAA CTCTAGTGAC TCTAGTGAGT

           ....|....| ....|....| ....|....| ....|....| ....|....| ....|....| ....|....| ....|....| ....|....| ....|....|
                 6810       6820       6830       6840       6850       6860       6870       6880       6890       6900
CRF07_BC   ATTATAGATT AATAAATTGT AATACCTCAG CCATAACACA AGCCTGTCCA AAGGTCACTT TTGATCCAAT TCCTATACAT TATTGCACTC CAGCTGGTTA

           ....|....| ....|....| ....|....| ....|....| ....|....| ....|....| ....|....| ....|....| ....|....| ....|....|
                 6910       6920       6930       6940       6950       6960       6970       6980       6990       7000
CRF07_BC   TGCAATTCTC AAGTGTAATG ATAAAAAATT CAATGGGACA GGACCATGCT CTAATGTTAG CACAGTACAA TGTACACATG GGATTAAGCC AGTGGTATCA

           ....|....| ....|....| ....|....| ....|....| ....|....| ....|....| ....|....| ....|....| ....|....| ....|....|
                 7010       7020       7030       7040       7050       7060       7070       7080       7090       7100
CRF07_BC   ACTCAACTAC TGTTAAATGG TAGCCTAGCA GAAGGAGAAA TAATAATTAG ATCTGAAAAT CTGACAAACA ATGCCAAAAC GATAATAGTA CATCTTAATC

           ....|....| ....|....| ....|....| ....|....| ....|....| ....|....| ....|....| ....|....| ....|....| ....|....|
                 7110       7120       7130       7140       7150       7160       7170       7180       7190       7200
CRF07_BC   AATCTGTAGA AATTGTATGT ACAAGACCCG GCAATAATAC AAGAAAAAGT ATAAGGATAG GACCAGGACA AACATTTTAT GCAACAGGAG ATATAATAGG

           ....|....| ....|....| ....|....| ....|....| ....|....| ....|....| ....|....| ....|....| ....|....| ....|....|
                 7210       7220       7230       7240       7250       7260       7270       7280       7290       7300
CRF07_BC   AGACATAAGA CAAGCACATT GTAACATTAG TGCAGGAAAT TGGAATGAAA CTTTACACAG AGTAAGTAAA AAATTAACAG AACATTTCCC GAATAAAACA

           ....|....| ....|....| ....|....| ....|....| ....|....| ....|....| ....|....| ....|....| ....|....| ....|....|
                 7310       7320       7330       7340       7350       7360       7370       7380       7390       7400
CRF07_BC   ATAAAATTTG CATCATCCTC AGGAGGGGAC CTAGAAATTA CAACACATAG CTTTAATTGT AGAGGAGAAT TTTTCTATTG TAATACATCA GGCCTGTTTA

           ....|....| ....|....| ....|....| ....|....| ....|....| ....|....| ....|....| ....|....| ....|....| ....|....|
                 7410       7420       7430       7440       7450       7460       7470       7480       7490       7500
CRF07_BC   ATGGTACATA CATGTTTAAT GGTACAAGGG GTAATTCAAG CTCAAACTCA ACCATCACAA TCCCATGCAG AATAAAGCAA ATTATAAATA TGTGGCAGGA

           ....|....| ....|....| ....|....| ....|....| ....|....| ....|....| ....|....| ....|....| ....|....| ....|....|
                 7510       7520       7530       7540       7550       7560       7570       7580       7590       7600
CRF07_BC   GGTAGGACGA GCAATGTATG CCCCTCCCAT TGCAGGAAAC ATAACATGTA AATCAAATAT CACAGGGCTA CTATTGGTAC GTGATGGAGG GCCAGATGAT

           ....|....| ....|....| ....|....| ....|....| ....|....| ....|....| ....|....| ....|....| ....|....| ....|....|
                 7610       7620       7630       7640       7650       7660       7670       7680       7690       7700
CRF07_BC   ACAAAGAATG GTACAGAGAC ATTCAGACCT GGAGGAGGAG ATATGAGGGA CAATTGGAGA AGTGAATTAT ATAAATATAA AGTGGTAGAA ATTAAGCCAT

           ....|....| ....|....| ....|....| ....|....| ....|....| ....|....| ....|....| ....|....| ....|....| ....|....|
                 7710       7720       7730       7740       7750       7760       7770       7780       7790       7800
CRF07_BC   TGGGAGTAGC ACCCACTGCA GCAAAAAGGA GAGTGGTGGA GAGAGAAAAA AGAGCAGTGG GAATAGGAGC TGTGTTCCTT GGGTTCTTGG GAGCAGCAGG
```

```
          ....|....| ....|....| ....|....| ....|....| ....|....| ....|....| ....|....| ....|....| ....|....| ....|....|
                 7810       7820       7830       7840       7850       7860       7870       7880       7890       7900
CRF07_BC  AAGCACTATG GGCGCGGCGT CAATAACGCT GACGGTACAA GCCAGACAAT TGCTGTCTGG TATAGTGCAA CAGCAAAGCA ATTTGCTGAA GGCTATAGAG

          ....|....| ....|....| ....|....| ....|....| ....|....| ....|....| ....|....| ....|....| ....|....| ....|....|
                 7910       7920       7930       7940       7950       7960       7970       7980       7990       8000
CRF07_BC  GCGCAACAGC ATCTGTTGCA ACTCACGGTC TGGGGCATTA AGCAGCTCCA GACAAGAGTC CTGGCTATAG AAAGATACCT AAAGGATCAA CAGCTCCTAG

          ....|....| ....|....| ....|....| ....|....| ....|....| ....|....| ....|....| ....|....| ....|....| ....|....|
                 8010       8020       8030       8040       8050       8060       8070       8080       8090       8100
CRF07_BC  GGATTTGGGG CTGCTCTGGA AAACTCATCT GCACTACTGC TGTACCTTGG AACTCCAGTT GGAGTAACAA AACTCAAGAA GAGATTTGGG ATAACATGAC

          ....|....| ....|....| ....|....| ....|....| ....|....| ....|....| ....|....| ....|....| ....|....| ....|....|
                 8110       8120       8130       8140       8150       8160       8170       8180       8190       8200
CRF07_BC  CTGGATGCAG TGGGATAAAG AAATTAGTAA TTACACAAAC ACAATATACA GGTTGCTTGA AGACTCGCAA AACCAGCAGG AAAGGAATGA AAAAGATCTA

          ....|....| ....|....| ....|....| ....|....| ....|....| ....|....| ....|....| ....|....| ....|....| ....|....|
                 8210       8220       8230       8240       8250       8260       8270       8280       8290       8300
CRF07_BC  TTAGCATTGG ACAGTTGGAA AAATCTATGG AGTTGGTTTG ACATAACAAA TTGGCTGTGG TATATAAAAA TATTCATAAT GATAGTAGGA GGCTTGATAG

          ....|....| ....|....| ....|....| ....|....| ....|....| ....|....| ....|....| ....|....| ....|....| ....|....|
                 8310       8320       8330       8340       8350       8360       8370       8380       8390       8400
CRF07_BC  GTTTAAGAAT AATTTTTGCT GTGCTCTCTA TAGTGAATAG AGTTAGGCAG GGATACTCAC CTTTGTCGTT TCAGACCCGT ATCCCGAACC CAGGGGGACC

          ....|....| ....|....| ....|....| ....|....| ....|....| ....|....| ....|....| ....|....| ....|....| ....|....|
                 8410       8420       8430       8440       8450       8460       8470       8480       8490       8500
CRF07_BC  CGACAGGCTC GGAAGAATCG AAGAAGAAGG TGGAGAGCAA GACAAAGCCA GATCCATTCG ATTAGTGAGC GGATTCTTAG CACTTGCCTG GGACGATCTG

          ....|....| ....|....| ....|....| ....|....| ....|....| ....|....| ....|....| ....|....| ....|....| ....|....|
                 8510       8520       8530       8540       8550       8560       8570       8580       8590       8600
CRF07_BC  CGGAACCTGT GCCTCTTCAG CTACCACCGA TTGAGAGACT TCATATTAGT GACAGCGAGA GTGGTGGAAC TTCTGGGACG CAGCAGTCTC AGGGGACTAC

          ....|....| ....|....| ....|....| ....|....| ....|....| ....|....| ....|....| ....|....| ....|....| ....|....|
                 8610       8620       8630       8640       8650       8660       8670       8680       8690       8700
CRF07_BC  AGACGGGGTG GGAAGCCCTT AAATATCTGG GAAGTCTTGT GCAGTACTGG GGTCAGGAGC TAAAAAAGAG TGCTATTAGT CTGATTGATA CCATAGCAAT

          ....|....| ....|....| ....|....| ....|....| ....|....| ....|....| ....|....| ....|....| ....|....| ....|....|
                 8710       8720       8730       8740       8750       8760       8770       8780       8790       8800
CRF07_BC  AGCAGTAGCT GAAGGAACAG ATAGGATTAT AGAATTAGTA CAAGGACTTT GTAGAGCTAT CTACAACATA CCTACAAGAG TAAGACAGGG CTTGGAAGCA

          ....|....| ....|....| ....|....| ....|....| ....|....| ....|....| ....|....| ....|....| ....|....| ....|....|
                 8810       8820       8830       8840       8850       8860       8870       8880       8890       8900
CRF07_BC  GCTTTGCAAT AAAATGGGGG CTAAGTGGTC AAAAAGTAGC ATAGTTGGAT GGCCTGCTGT AAGGGAGAGA ATAAGACGAA CTGAGCCAGC GGCAGATGGG

          ....|....| ....|....| ....|....| ....|....| ....|....| ....|....| ....|....| ....|....| ....|....| ....|....|
                 8910       8920       8930       8940       8950       8960       8970       8980       8990       9000
CRF07_BC  GTGGGAGCAG TATCTCGAGA CCTGGAAAAA CATGGAGCAA TCACAAGTAG CAATACAGCA GCTACTAATG CTGATTGTGC CTGGCTGGAA GCACAAGAGG

          ....|....| ....|....| ....|....| ....|....| ....|....| ....|....| ....|....| ....|....| ....|....| ....|....|
                 9010       9020       9030       9040       9050       9060       9070       9080       9090       9100
CRF07_BC  AGGAAGAGGA GGTGGGTTTT CCAGTCAGAC CTCAGGTACC TTTAAGACCA ATGACTTACA AGGGAGCTCT AGATCTTAGC TTCTTTTTAA AAGAAAAGGG

          ....|....| ....|....| ....|....| ....|....| ....|....| ....|....| ....|....| ....|....| ....|....| ....|....|
                 9110       9120       9130       9140       9150       9160       9170       9180       9190       9200
CRF07_BC  GGGACTGGAA GGGTTAATTT ACTCTAAGAA AAGGCAAGAG ATCCTTGATT TGTGGGTCTA TCACACTCAA GGCTACTTCC CTGATTGGCA CAACTACACA

          ....|....| ....|....| ....|....| ....|....| ....|....| ....|....| ....|....| ....|....| ....|....| ....|....|
                 9210       9220       9230       9240       9250       9260       9270       9280       9290       9300
CRF07_BC  CCAGGACCAG GGGTCAGATT TCCACTGACT TTTGGGTGGT GCTTCAAGCT AGTACCAGTT GATCCAAAGG AAGTAGAAGA GGCCAACGAA GGAGAAGACA

          ....|....| ....|....| ....|....| ....|....| ....|....| ....|....| ....|....| ....|....| ....|....| ....|....|
                 9310       9320       9330       9340       9350       9360       9370       9380       9390       9400
CRF07_BC  ACAGCTTGCT ACACCCTGTG TGCCAGCATG GAATGGATGA TGAACACAGA GAAGTGTTAA AGTGGAAGTT TGACAGTCAC CTAGCACACA GCCACAAGGC

          ....|....| ....|....| ....|....| ....|....| ....|....| ....|....| ....|....| ....|....| ....|....| ....|....|
                 9410       9420       9430       9440       9450       9460       9470       9480       9490       9500
CRF07_BC  CCGCGAGCTA CATCCGGAGT TTTACAAAGA CTGCTGACAC AGAAGGGACT TTCCGCAGGG ACTTTCCACT GGGGCGTTCT AGGAGGTGTG GTCTGGGCGG

          ....|....| ....|....| ....|....| ....|....| ....|....| ....|....| ....|....| ....|....| ....|....| ....|....|
                 9510       9520       9530       9540       9550       9560       9570       9580       9590       9600
CRF07_BC  GACTGGGAGT GGTCAACCCT CAGATGCTGC ATATAAGCAG CTGCTTTTCG CCTGTACTGG GTCTCTCTAG TTAGACCAGA TCTGAGCCTG GGAGCTCTCT

          ....|....| ....|....| ....|....| ....|....| ....|....| ....|....| ....|....| ....|....| ....|....| ....|....|
                 9610       9620       9630       9640       9650       9660       9670       9680       9690       9700
CRF07_BC  GGCTGACTAG GAAACCCACT GCTCAAGCCT CAATAAAAGC TTGCCTTGAG TGCTACAAGT AGTGTGTGCC CGTCTGTTGT GTGACTCTGG TAACTAGAGA

          ....|....| ....|..
                 9710
CRF07_BC  TCCCTCAGAC CACGCGT
```

Figure S2. The full-length sequence of the constructed HIV-1 CRF07_BC infectious clone

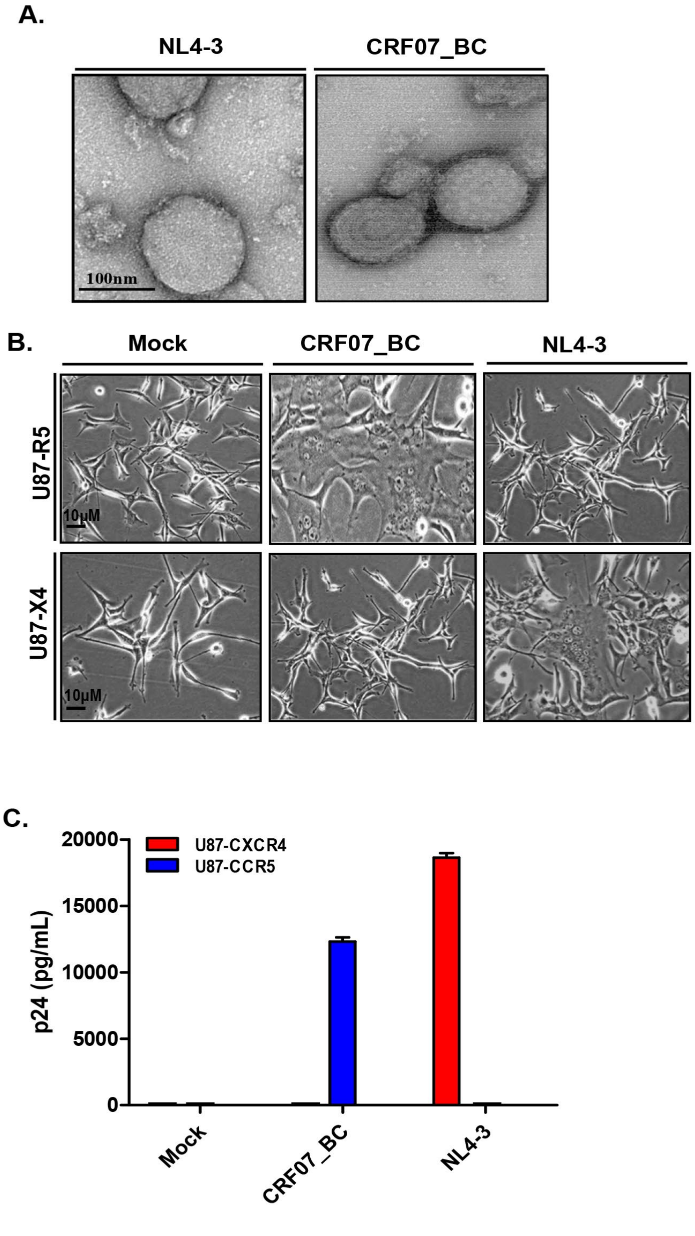

Figure S3. Determination of viral characteristics of HIV-1 CRF07_BC viruses. (A) The pNL4-3 and pCRF07_BC were transfected into HEK293T cells and the viral supernatants were subjected to TEM observation. (B) For determination of virus tropism, the HIV-1 CRF07_BC and subtype NL4-3 viruses were administered to U87-R5 (upper panel) and U87-X4 cells (lower panel), and the virus-induced CPE of the cells was observed. (C) The viral supernatants collected from (B) were subjected to HIV-1 p24 determination.

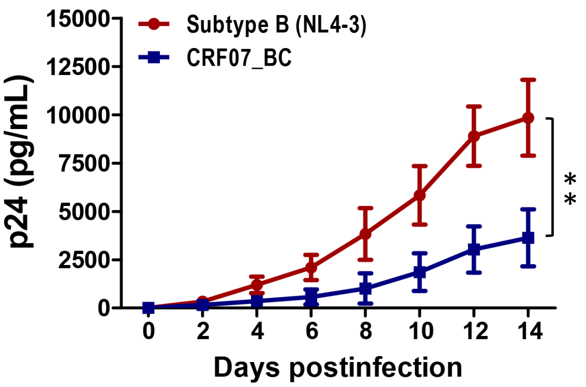

Figure S4. Viral characteristics of HIV-1 CRF07_BC. Viral growth kinetics was conducted using Jurkat-R5 cells (the Jurkat-CXCR4-CCR5 stably expressing cells) infected with B subtype NL4-3 and CFR07_BC viruses. The viral supernatants were collected at different time points and subjected to HIV-1 p24 determination. Quantitative data represent the mean ± SD of results from three independent experiments (**P < 0.01).

Binding Constant	Galectin-3
Ka (M^{-1} S^{-1})	$4.567x10^{-4}$
Kd (S^{-1})	$3.223x10^{-4}$
KD(M)	$7.073x10^{-9}$

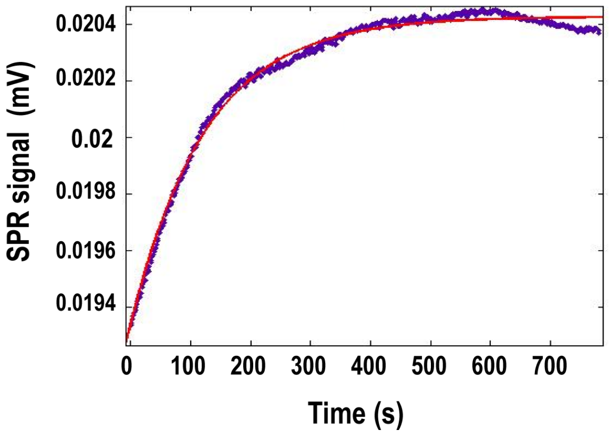

Figure S5: Determination of the binding affinities between galectin-3 and CRF07_BC gp120. The binding affinities between Gal3 and HIV-1 CRF07_BC gp120 were measured via SPR. The dissociation constant (KD) of galectin-3 was determined.

Table S1. The genotyping of enrolled HIV-1(+) IDUs

**Sample ID**	***Genotyping***		***Consistency***
	Multiplex PCR	***Sequencing & Phylogenetic analysis***	
TWKMU_IDU_001	CRF07_BC	CRF07_BC	Yes
TWKMU_IDU_002	CR07_BC	CR07_BC	Yes
TWKMU_IDU_003	B	B	Yes
TWKMU_IDU_004	CRF07_BC	CRF07_BC	Yes
TWKMU_IDU_005	CRF07_BC	CRF07_BC	Yes
TWKMU_IDU_006	CRF07_BC	CRF07_BC	Yes
TWKMU_IDU_007	CRF07_BC	CRF07_BC	Yes
TWKMU_IDU_008	CRF07_BC	CRF07_BC	Yes
TWKMU_IDU_009	CRF07_BC	CRF07_BC	Yes
TWKMU_IDU_010	CRF07_BC	CRF07_BC	Yes
TWKMU_IDU_011	CRF07_BC	CRF07_BC	Yes
TWKMU_IDU_012	B	B	Yes
TWKMU_IDU_013	CRF07_BC	CRF07_BC	Yes
TWKMU_IDU_014	B	B	Yes
TWKMU_IDU_015	CRF07_BC	CRF07_BC	Yes
TWKMU_IDU_016	CRF07_BC	CRF07_BC	Yes
TWKMU_IDU_017	CRF07_BC	CRF07_BC	Yes
TWKMU_IDU_018	CRF01_AE	CRF01_AE	Yes
TWKMU_IDU_019	CRF07_BC	CRF07_BC	Yes
TWKMU_IDU_020	CRF07_BC	CRF07_BC	Yes
TWKMU_IDU_021	CRF07_BC	CRF07_BC	Yes
TWKMU_IDU_022	CRF07_BC	CRF07_BC	Yes
TWKMU_IDU_023	CRF07_BC	CRF07_BC	Yes
TWKMU_IDU_024	CRF07_BC	CRF07_BC	Yes
TWKMU_IDU_025	CRF07_BC	CRF07_BC	Yes
TWKMU_IDU_026	CRF07_BC	CRF07_BC	Yes
TWKMU_IDU_027	CRF07_BC	CRF07_BC	Yes
TWKMU_IDU_028	CRF07_BC	CRF07_BC	Yes
